# Numerical Modelling of Effects of Biphasic Layers of Corrosion Products to the Degradation of Magnesium Metal In Vitro

**DOI:** 10.3390/ma11010001

**Published:** 2017-12-21

**Authors:** Safia K. Ahmed, John P. Ward, Yang Liu

**Affiliations:** 1 Department of Mathematical Sciences, Loughborough University, Loughborough LE11 3TU, UK; S.K.Ahmed@lboro.ac.uk; 2 Department of Mechanical, Electrical and Manufacturing Engineering, Loughborough University, Loughborough LE11 3TU, UK; Y.Liu3@lboro.ac.uk

**Keywords:** magnesium, corrosion, mathematical model, moving boundaries, numerical solution

## Abstract

Magnesium (Mg) is becoming increasingly popular for orthopaedic implant materials. Its mechanical properties are closer to bone than other implant materials, allowing for more natural healing under stresses experienced during recovery. Being biodegradable, it also eliminates the requirement of further surgery to remove the hardware. However, Mg rapidly corrodes in clinically relevant aqueous environments, compromising its use. This problem can be addressed by alloying the Mg, but challenges remain at optimising the properties of the material for clinical use. In this paper, we present a mathematical model to provide a systematic means of quantitatively predicting Mg corrosion in aqueous environments, providing a means of informing standardisation of in vitro investigation of Mg alloy corrosion to determine implant design parameters. The model describes corrosion through reactions with water, to produce magnesium hydroxide Mg(OH)${}_{2}$, and subsequently with carbon dioxide to form magnesium carbonate MgCO${}_{3}$. The corrosion products produce distinct protective layers around the magnesium block that are modelled as porous media. The resulting model of advection–diffusion equations with multiple moving boundaries was solved numerically using asymptotic expansions to deal with singular cases. The model has few free parameters, and it is shown that these can be tuned to predict a full range of corrosion rates, reflecting differences between pure magnesium or magnesium alloys. Data from practicable in vitro experiments can be used to calibrate the model’s free parameters, from which model simulations using in vivo relevant geometries provide a cheap first step in optimising Mg-based implant materials.

## 1. Introduction

Magnesium is a biodegradable, lightweight structured metal that is becoming increasingly popular for orthopaedic implants due to its desirable properties. It is an essential element in the human body for providing normal neurological and muscular functions [1,2], and is detected in large amounts in the bone tissue. It is reported in [3,4,5] that magnesium alloys have a Young’s modulus and specific density closer to that of the human bone than the frequently used non-biodegradable titanium and stainless steel implants; this eliminates the problem of stress shielding. Furthermore, the nature of a biodegradable implant saves on expenses and risks associated with undergoing a second surgery to remove the hardware. Biodegradable materials that are typically used in the bone implant industry are polymers and ceramics, which are not as sturdy as metal implants and, consequently, their applications are limited [4], thus highlighting the advantages of a biodegradable metal implant, such as magnesium.

While there are numerous benefits of magnesium implants, in its pure form, magnesium corrodes rapidly in an aqueous environment, which is consistently an obstacle for biomaterial scientists [6]. This corrosion is due to magnesium being reactive to water to form magnesium hydroxide, releasing bubbles of hydrogen that can accumulate to form gas pockets near the implant [4]. Furthermore, the rapid corrosion causes the loss of mechanical support before the newly formed bone tissue can bear the load, thereby preventing the bone from healing correctly. These limitations can be mitigated using alloys of magnesium (e.g., using calcium, zinc, rare earths, etc. [7,8,9]), and research into this for the use in orthopaedic implant devices is vastly growing [10,11,12]. Physiological consequences of magnesium implants has also been explored, including the blood and organ compositions of corrosion products [13] and collagen interaction with implants [14]. However, the major challenge that continues to be faced by regulation and industry is how to predict the corrosion rate of magnesium metal-based biomaterials in vitro and correlate to the timing of its absorption in vivo. In this work, the problem is approached using mathematical modelling aimed to provide a systematic means of quantitatively describing the physiochemical interaction during magnesium corrosion processes in vitro, further informing standardisation of in vitro investigation of magnesium alloy corrosion and implant design parameters for optimal bone growth.

The application of mathematical modelling in metal corrosion has been studied widely for some time [15,16,17,18]. Our approach employs a porous media extension of a model that is used to describe atmospheric corrosion of a block of copper [16,19]; their model is built upon the chemical reactions incurred as the copper sample corrodes using an advection–diffusion system. There are numerous studies of similar problems for a range of metals that neglect the advective contribution to the corrosion dynamics, resulting in Stefan-like problems to describe corrosion in, for example, zirconium [20] and steel [21]. Magnesium corrosion has been the subject of a small number of modelling studies [22,23,24]. In [23], a simple two-phase bulk model of magnesium corrosion was proposed and parameter fitted to experimental data; however, the model is not explicit in the products of corrosion. A spatially explicit model of a galvanised magnesium was proposed in [24]; here, the magnesium block was a fixed domain and they showed that the thickness of the galvanised layer affected corrosion. The authors of [22] used a level-set approach to describe moving interfaces separating a pure magnesium block and a partially corroded phase consisting of dissolved Mg ions (magnesium chloride) and a protective layer of magnesium hydroxide.

In aqueous and in physiological relevant environments, carbon dioxide and dissolved bicarbonates can react with magnesium hydroxide to ultimately form magnesium carbonate, which is largely insoluble and forms a robust protective layer around the magnesium block. This latter feature is currently absent in magnesium corrosion models and is believed to play an important part in the performance of an implant in vivo. In this paper, we adopted the approach of [16], but applied it to magnesium and the resulting layers of corrosive products Mg(OH)_2_ and MgCO_3_; this is the first study to consider the latter product in a magnesium corrosion model. A further novelty is to consider these layers as porous media, whereby there is fluid phase flow within the pores of the developing crystal structures, so that the reactants H_2_O and CO_2_ can advect, as well as diffuse, through them. Whether or not this porous media assumption leads to substantially different results will be one of the aspects explored in this paper. An aim is to guide relatively simple in vitro experimentation that can inform the model parameters, which can then be used in the modelling of magnesium in more clinically relevant environments, with more appropriate geometries and dimensions, to predict corrosion in vivo.

In the next section, a partial differential equation (PDE) model is developed as an advection–diffusion system, whereby magnesium is assumed to corrode through a series of chemical reactions in vitro. The model is simplified and non-dimensionalised in preparation for the numerical investigations described in Section 3. In the final sections, the main results are discussed and concluded.

## 2. Mathematical Model

In vitro, either in water or a clinically relevant media, the magnesium component of a proposed implant will initially corrode through the reaction with water, according to

(1) $$\begin{array}{c} Mg+2H_{2}O⟶Mg(OH{)}_{2}+H_{2} \end{array},$$

leading to the production of hydrogen and a protective layer of magnesium hydroxide. The hydrogen gas evolves from the solution leaving the hydroxide film on the magnesium surface, which is only stable at a high pH and, in physiological environments, is vulnerable to further corrosion [11]. In water, dissolved CO_2_ reacts with Mg(OH)_2_ to ultimately form magnesium carbonate in a reaction summarised by [25,26],

(2) $$\begin{array}{c} Mg(OH{)}_{2}+CO_{2}⟶MgCO_{3}+H_{2}O \end{array},$$

which is used as the basis for the model formulation. In water, the principle reacting agent with Mg(OH)_2_ are hydrogen carbonates ions, HCO_3_^−^, formed from the reaction of dissolved CO_2_ with water. The reaction of these ions with Mg(OH)_2_ leads to the formation of magnesium hydrogen carbonate, Mg(HCO_3_)_2_, which then decomposes to form magnesium carbonate, MgCO_3_. The intermediate magnesium hydrogen carbonate is thermodynamically unstable at atmospheric levels of CO_2_ [26] and demonstrated experimentally [7,27]; we thus assume the intermediate hydrogen carbonate form is short-lived and will therefore be neglected in the modelling. A more realistic representation of the overall reaction is [7],

(3) $$\begin{array}{ccc} CO_{2}+H_{2}O & \;⟷\; & HC{O_{3}}^{-}H^{+}, \end{array}$$

(4) $$\begin{array}{ccc} \begin{array}{c} Mg(OH{)}_{2}+HC{O_{3}}^{-} \end{array} & \begin{array}{c} ⟶ \end{array} & \begin{array}{c} MgCO_{3}+H_{2}O+OH^{-} \end{array}. \end{array}$$

In more clinically relevant environments, for example using cell culture medium in vitro, the presence of a hydrogen carbonate buffering system using concentrations reflecting that in the blood (27 mmol/L) [28] means that HCO_3_^−^ will lead to the corrosion of Mg(OH)_2_ via the reaction in Equation (4). The formulation of the model with regards to conversion of the hydroxide to carbonate forms means that both reactions Equations (2) and (4) are described, and the variable $C_{2}$ in this model can be viewed either as the concentration of CO_2_ or HCO_3_− or both as the stoichiometry for water is the same; for simplicity, the discussion on Mg(OH)_2_ corrosion in the remainder of this paper will refer to CO_2_ and the reaction in Equation (2). We note that the resulting layer of magnesium carbonate is more stable and has been proposed as a layer to delay the corrosion process [25]. We further assume in the model that, throughout the corrosion process of Mg, the environment is stable (e.g., pH is unchanged, as would be expected in a buffered medium in vitro) and that supplies of water and CO_2_ are inexhaustible.

**Figure 1:**
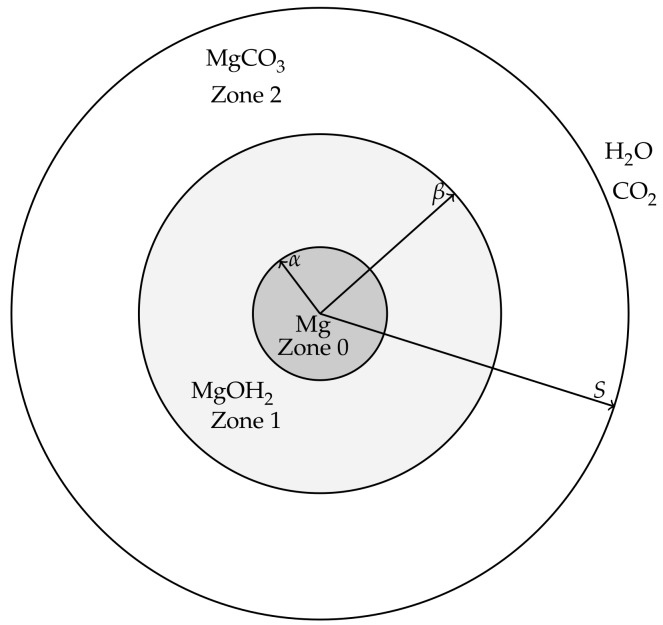
A physiochemical schematic of the magnesium corrosion system used in the model for cylindrical and spherical geometries. The pure magnesium or magnesium alloy exists in the core (Zone 0, 0≤r<α(t)), the magnesium hydroxide forms a middle layer (Zone 1, α(t)<r<β(t)) and the outer layer consists of magnesium carbonate (Zone 2, β(t)<r<S(t)).

The modelling is aimed at describing events from the beginnings of the corrosion process of a magnesium or magnesium alloy pellet, through the formation of hydroxide and carbonate layers, until the magnesium and magnesium hydroxide is exhausted and only the carbonate remains. For simplicity, we will refer to the magnesium (alloy) pellet as “pure magnesium” to distinguish it from the hydroxide and carbonate forms. The magnesium pellet is assumed to be non-porous and corrodes in a way that maintains a smooth surface, i.e., surface pitting and cracking is assumed negligible at leading order. It is therefore envisioned that, in the corroding process, the magnesium is surrounded by a layer of Mg(OH)_2_, which in turn is surrounded by a layer of MgCO_3_ (see Figure 1). In order for the magnesium pellet to corrode further, water must be able to diffuse though the carbonate and hydroxide layers to react at the pellet’s surface and CO_2_ must be able to diffuse through the carbonate layer to reach the hydroxide compound interface. These assumptions lead to a model that describes both the transport and reaction processes of water and carbon dioxide as well as the location of the interfaces between magnesium and its compounds, which are deposited on the surface of the magnesium metal as corrosion products. The modelling will be formulated for a general 1D geometry, namely Cartesian (describing a magnesium slab), cylindrical (a magnesium rod) and spherical geometry (a magnesium ball). The hydroxide and carbonate layers are treated as porous media, thereby the movement speed, $v_{s_{i}}$, of the “solid” components, i.e., the Mg(OH)_2_ and MgCO_3_, is distinct to that of the fluid and dissolved gas components, i.e., H_2_O and CO_2_, namely $v_{f}$; this is a novel feature in metal corrosion models. Fortunately, by assuming ideal geometries, a closed system of equations can be derived based on mass conservation alone.

### 2.1. Mathematical Modelling

Figure 1 shows a cross-section of the cylindrical/spheroid scenarios for the model; the Cartesian case simply has three layers bounded by parallel, linear interfaces. Applying the above ideal geometries means that the resulting model will only consider changes in one-spatial dimension. Writing *r* and *t* as the spatial coordinate and time variable, respectively, we denote the coordinates of the moving interfaces as follows:

- r=α(t) is the location of the magnesium to magnesium hydroxide interface.
- r=β(t) is the location of the magnesium hydroxide to magnesium carbonate interface.
- r=S(t) is the location of outer edge, exposed to concentrations of water and carbon dioxide, representative of the in vitro environment containing HCO_3_^−^/CO_2_ buffering system.

We also denote the spaces between the interfaces as

- Zone 0: the Mg layer r<α(t),
- Zone 1: the Mg(OH)_2_ layer α(t)<r<β(t),
- Zone 2: the MgCO_3_ layer β(t)<r<S(t).

The movement of the magnesium compounds, which is when the hydroxide deposits on the magnesium surface and when the carbonate produces on the hydroxides surface, are denoted by velocities $v_{s_{i}}$, where i=1,2 corresponding to the zone; and the flow of water and carbon dioxide in the fluid phases are denoted by velocities $v_{f_{i}}$.

It is assumed that the solid structure of Zones 0–2 is homogeneous, i.e., they consist of a fixed volume fraction of the magnesium compound and non-traversable space ($\varepsilon_{i}$) and traversable space ($1-\varepsilon_{i}$); traversable space is defined to be continuous channels of space and excludes completely enclosed gaps in the solid structure. In the magnesium layer, it is assumed that it is entirely non-traversable, hence $\varepsilon_{0}=1$. All reactions are assumed to occur only at the interfaces α(t) and β(t), whereby the rate at which these interfaces move depends on the local rate of reaction. Considering the availability of inexhaustible CO_2_ in vitro, we note that in the physiochemical representative corrosion system, the magnesium and magnesium hydroxide regions will eventually vanish, i.e., α=0 and β=0, respectively; consequently, there are distinct time phases in the corrosion process that need to be separately handled by the model. We define $t=T_{\alpha }$ as the point in time when α(t)=0 (i.e., α(t)>0 for $t<T_{\alpha }$), and likewise $t=T_{\beta }$ for when β(t)=0. Once β=0, i.e., for $t>T_{\beta }$, there are no further developments in the system and all that remains is a block of magnesium carbonate.

With $v_{s_{1}}\left(r,t\right)$ and $v_{s_{2}}\left(r,t\right)$ being the radial velocities of the solid phases, the conservation of mass implies

(5) $$\begin{array}{ccc} \frac{1}{r^{d}}\frac{\partial }{\partial r}\left(r^{d}\varepsilon_{1}\;v_{s_{1}}\right) & = & 0\;\;\;\;\;\;\;r\in (\alpha ,\beta ), \end{array}$$

(6) $$\begin{array}{ccc} \frac{1}{r^{d}}\frac{\partial }{\partial r}\left(r^{d}\varepsilon_{2}\;v_{s_{2}}\right) & = & 0\;\;\;\;\;\;\;r\in (\beta ,S), \end{array}$$

where d=0,1,2 represent Cartesian, cylindrical and spherical geometry, respectively. Here, $\varepsilon_{1}$ and $\varepsilon_{2}$ are the solid phase volume fractions, respectively; we note that $\varepsilon_{1}$ and $\varepsilon_{2}$ are constant in their respective zones so can be divided out; however, the fraction term will be retained in the model derivation for completeness. We will for simplicity assume that the fluid phase consists of all non-solid materials and that it is non-compressible. Conservation of total material volume implies that

$$\begin{array}{c} \frac{1}{r^{d}}\frac{\partial }{\partial r}\left(r^{d}\left(\varepsilon_{i}\;v_{s_{i}}+\left(1-\varepsilon_{i}\right)\;v_{f_{i}}\right)\right)\;=\;0, \end{array}$$

for i=1,2; hence, using Equations (5) and (6), we have

(7) $$\begin{array}{ccc} \frac{1}{r^{d}}\frac{\partial }{\partial r}\left(r^{d}\left(1-\varepsilon_{1}\right)\;v_{f_{1}}\right) & = & 0\;\;\;\;\;\;\;r\in (\alpha ,\beta ), \end{array}$$

(8) $$\begin{array}{ccc} \frac{1}{r^{d}}\frac{\partial }{\partial r}\left(r^{d}\left(1-\varepsilon_{2}\right)\;v_{f_{2}}\right) & = & 0\;\;\;\;\;\;\;r\in (\beta ,S). \end{array}$$

We emphasise that the flow here does not encompass that of the fluid/gas trapped in non-traversable pores in the solid structure.

It is assumed that water can be transported via diffusion and advection throughout Zones 1 and 2, whilst carbon dioxide is limited to Zone 2; carbon dioxide is assumed to be immediately exhausted on contact with Mg(OH)_2_ on the r=β interface (see Section 2.1.1). Let $W_{1}$ be the mass concentration of water in the pores of Mg(OH)_2_ structure and $W_{2}$ and $C_{2}$ be that of water and carbon dioxide, respectively, in the MgCO_3_. The transport equations for the water and carbon dioxide are

(9) $$\begin{array}{cc} \frac{\partial (\left(1-\varepsilon_{1}\right)W_{1})}{\partial t}\;=\;-\frac{1}{r^{d}}\frac{\partial }{\partial r}\left(r^{d}\left(1-\varepsilon_{1}\right)J_{W_{1}}\right)\;\;\;\; & r\in (\alpha ,\beta ), \end{array}$$

(10) $$\begin{array}{cc} \left.\begin{array}{c} \frac{\partial (\left(1-\varepsilon_{2}\right)W_{2})}{\partial t}\;=\;-\frac{1}{r^{d}}\frac{\partial }{\partial r}\left(r^{d}\left(1-\varepsilon_{1}\right)J_{W_{2}}\right)\\ \frac{\partial (\left(1-\varepsilon_{2}\right)C_{2})}{\partial t}\;=\;-\frac{1}{r^{d}}\frac{\partial }{\partial r}\left(r^{d}\left(1-\varepsilon_{1}\right)J_{C_{2}}\right) \end{array}\right\} & r\in (\beta ,S), \end{array}$$

with the fluxes defined as $J_{X_{i}}=-D_{X}\partial X_{i}/\partial r+v_{f_{i}}X_{i}$; again with the $\varepsilon_{i}$s being constants, then the fluid fraction can be divided out in each of the equations. Here, $D_{X}$ is the diffusion coefficient with *X* representing water, *W*, or carbon dioxide, *C*.

#### 2.1.1. Boundary and Interface Conditions

It is assumed that the initial state consists only of a magnesium, and impose

(11) $$\begin{array}{c} t\;=\;0:\;\;\;\;\;S\;=\;\beta \;=\;\alpha \;=\;S_{0}, \end{array}$$

where the initial thickness or radius $S_{0}>0$. Water and carbon dioxide is sourced at the outer surface r=S, which moves at speed equal to the local velocity, hence

(12) $$\begin{array}{c} r\;=\;S\left(t\right):\;\;\;\;\;W_{2}=W_{0}^{*},\;\;C_{2}=C_{0}^{*},\;\;\dot{S}=v_{2}, \end{array}$$

where $\dot{S}=dS/dt$. The conditions on the interfaces are more complex and change at critical points of the corrosion process. On r=α(t), water reacts with the surface of the magnesium block. Assuming that the magnesium surface is uniform, then the rate of reaction, $R_{\alpha }$, will be dependent on the water concentration and flux there. As two water molecules are consumed, we assume by the law of mass action applied to Equation (1) that $R_{\alpha }=kW_{1}^{2}$, where *k* is a rate constant. There are two cases that will be considered in this paper:

- *Case 1* considers the limit k→∞, where the reaction is so rapid that water is immediately exhausted on r=α(t) interface, hence $W_{1}=0$ here. This assumption is most consistent with that used for carbon dioxide on r=β(t). The boundary conditions Equations (13) and (15) are relevant.
- *Case 2* considers k<∞, whereby $W_{1}>0$ on r=α(t). In a short time, whereby 1−β(t)/S(t)≪1, the small distance for the carbon dioxide to diffuse means that Mg(OH)_2_ immediately becomes exhausted on production and Mg converts to MgCO_3_, in effect, immediately; consequently, α(t)=β(t) during this transient. In time, the thickness of Zone 2, S−β, becomes sufficiently large for the reaction to exhaust the carbon dioxide on r=β, allowing the Mg(OH)_2_ layer to grow. Let $t=T_{\alpha =\beta }$ be the smallest time at which $C_{2}\left(\beta \left(t\right),t\right)=0$; then, for $t<T_{\alpha =\beta },$ the conditions in Equation (16) hold, whilst, for $t>T_{\alpha =\beta }$, Equations (13) and (15) are then imposed.

In both cases, the Mg will eventually be exhausted and the conditions in Equations (14) and (15) are then relevant.

Let *A* be the area of a surface element on a magnesium surface; then, the volume change rate upon this element of the Mg block is $A\dot{\alpha }$, translating to a molar change rate of $\mu_{0}\dot{\alpha }A$ (see Table 1). Consequently, the water molar flux through r=α(t) as the boundary moves is $\left(1-\varepsilon_{1}\right)\left(-W_{1}\dot{\alpha }+J_{W_{1}}\right)A/M_{W}=2\mu_{0}\dot{\alpha }A$, since two molecules of water are consumed and noting constant $M_{W}$ is equal to mass/mol of water. For the k→∞ case, this provides the equation for the moving boundary α(t), whilst, for k<∞ we have in addition the mass flux through *A* satisfying $\left(1-\varepsilon_{1}\right)\left(-W_{1}\dot{\alpha }+J_{W_{1}}\right)A=-2R_{\alpha }$. From Table 1, the quantities $\rho_{i}$ and $\mu_{i}$ represent the values from the magnesium compounds deposited as layers of corrosion products, hence volume elements are inclusive of the void fraction. The volume fraction difference through converting Mg to Mg(OH)_2_ is $\omega_{\alpha }-1$, where $\omega_{\alpha }=\mu_{0}/\mu_{1}$; consequently, volume gain rate from the reaction yields $v_{s_{1}}A=-\left(\omega_{\alpha }-1\right)\dot{\alpha }A$, noting that $\omega_{\alpha }>1$ implies a gain in volume so that $v_{s_{1}}$ must have an opposite sign to $\dot{\alpha }$. The final condition results from a no slip condition to the fluid phase on α(t), i.e., $v_{f_{i}}=\dot{\alpha }$. In summary, the conditions are on

(13) $$\begin{array}{c} \begin{array}{cc} r\;=\;\alpha (t): & v_{s_{1}}=-\left(\omega_{\alpha }-1\right)\dot{\alpha },\;v_{f_{1}}=\dot{\alpha },\;\left(1-\varepsilon_{1}\right)\left(-\dot{\alpha }W_{1}+J_{W_{1}}\right)=2\frac{M_{W}}{M_{0}}\rho_{0}\dot{\alpha },\\ & W_{1}=0\;for\;k\to \infty \;\;\;\;or\;\;\;\;\frac{M_{W}}{M_{0}}\rho_{0}\dot{\alpha }\;=\;-kW_{1}^{2}\;for\;k<\infty . \end{array} \end{array}$$

These conditions hold for α(t) > 0. On exhaustion of the pure Mg block and α(t)≡0, the boundary conditions are

(14) $$\begin{array}{c} \begin{array}{cc} r\;=\;0: & v_{s_{1}}=0,\;v_{f_{1}}=0,\;J_{W_{1}}=0. \end{array} \end{array}$$

For the cases on *k* described above, and $t>T_{\alpha =\beta }$ for k<∞, then, on r=β, the carbon dioxide is assumed to be completely consumed by its reaction with Mg(OH)_2_, whilst a water molecule is produced; for the latter, we assume the concentration is continuous across the interface, i.e., $[W_{i}]=0$, where the shorthand $\left[W_{i}\right]=W_{2}-W_{1}$ is used below. Letting *A* be again the area of a surface element on r=β, then the rate of volume loss of Mg(OH)_2_ is $(v_{s_{1}}-\dot{\beta })A$ and the molar loss rate is therefore $R_{\beta }=\mu_{1}\left(v_{s_{1}}-\dot{\beta }\right)A$. Consequently, the difference in molar flux of the water is $\left[A\left(\dot{\beta }W_{i}-J_{W_{i}}\right)\right]/M_{W}=R_{\beta }$, and carbon dioxide is $A\left(\dot{\beta }W_{i}-J_{W_{i}}\right)/M_{C}=-R_{\beta }$. The volume fraction difference from the reaction is $\omega_{\beta }-1$, by definition, and thus the volume gain rate $A(v_{s_{2}}-v_{s_{1}})$ is equal to $\left(\omega_{\beta }-1\right)\left(v_{s_{1}}-\dot{\beta }\right)A$. Conservation of fluid flux across the interface leads to $[\left(1-\varepsilon_{i}\right)\left(\dot{\beta }-v_{f_{i}}\right)]=0$. The conditions are on

(15) $$\begin{array}{c} \begin{array}{cc} r\;=\;\beta (t): & C_{2}=0,\;\;W_{2}=W_{1},\;\;v_{s_{2}}=v_{s_{1}}-\left(\omega_{\beta }-1\right)\left(\dot{\beta }-v_{s_{1}}\right),\\ & \left(1-\varepsilon_{1}\right)\left(v_{f_{1}}-\dot{\beta }\right)=\left(1-\varepsilon_{2}\right)\left(v_{f_{2}}-\dot{\beta }\right),\\ & \left(1-\varepsilon_{2}\right)\left(-\dot{\beta }W_{2}+J_{W_{2}}\right)-\left(1-\varepsilon_{1}\right)\left(-\dot{\beta }W_{1}+J_{W_{1}}\right)=-\frac{M_{W}}{M_{1}}\rho_{1}\left(\dot{\beta }-v_{s_{1}}\right),\\ & \left(1-\varepsilon_{2}\right)\left(-\dot{\beta }C_{2}+J_{C_{2}}\right)=\frac{M_{C}}{M_{1}}\rho_{1}\left(\dot{\beta }-v_{s_{1}}\right), \end{array} \end{array}$$

for β(t)>0.

For k<∞ and $t<T_{\alpha =\beta }$, in which Zone 1 is absent, the boundary conditions are

(16) $$\begin{array}{c} \begin{array}{cc} r\;=\;\alpha (t)=\beta (t): & v_{s_{2}}\;=\;-\left(\omega_{\alpha }\omega_{\beta }-1\right)\dot{\beta },\;v_{f_{2}}\;=\;\dot{\beta },\\ & \left(1-\varepsilon_{2}\right)\left(-W_{2}\dot{\beta }+J_{W_{2}}\right)\;=\;\frac{M_{W}}{M_{1}}\rho_{1}\dot{\beta },\;\;\;\;\frac{M_{W}}{M_{0}}\rho_{0}\dot{\beta }\;=\;-kW_{1}^{2},\\ & \left(1-\varepsilon_{2}\right)J_{C_{2}}=\frac{M_{C}}{M_{1}}\rho_{1}\dot{\beta }. \end{array} \end{array}$$

Here, the conversion of Mg to MgCO_3_ generates a volume fraction difference of $\omega_{\beta }\omega_{\alpha }-1$ and the stated condition on $v_{s_{1}}$ is equivalent to that in Equation (13) and likewise for $v_{f_{1}}$. The flux condition on water results from the net loss of one molecule from the overall reaction and likewise for CO_2_.

The current model assumes that MgCO_3_ will have no exit from the system, so the final state corresponds to when β=0, whereby all of the Mg and Mg(OH)_2_ have been exhausted.

Table 2 shows the boundary conditions used in Cases 1 and 2. Note, in the case k<∞ and $T_{\alpha }=T_{\alpha =\beta }$, Phases 2.1 and 2.3 are relevant. Conditions Equations (11) and (12) are relevant for both these cases on *k*.

### 2.2. Exact Solutions

Equations (5)–(8) are straightforward to integrate, though their solution depends on the various scenarios stated above. For Case 1, applying Equations (13) and (15) yields

(17) $$\begin{array}{c} \begin{array}{cc} v_{s_{1}}=-\frac{\left(\omega_{\alpha }-1\right)\;\dot{\alpha }\;\alpha^{d}}{r^{d}}, & \;\;v_{s_{2}}=-\frac{\omega_{\beta }\;\left(\omega_{\alpha }-1\right)\;\dot{\alpha }\;\alpha^{d}+\left(\omega_{\beta }-1\right)\;\dot{\beta }\;\beta^{d}}{r^{d}},\\ v_{f_{1}}=\frac{\dot{\alpha }\;\alpha^{d}}{r^{d}}, & v_{f_{2}}=\frac{\left(1-\varepsilon_{1}\right)\;\dot{\alpha }\;\alpha^{d}-\left(\varepsilon_{2}-\varepsilon_{1}\right)\;\dot{\beta }\;\beta^{d}}{\left(1-\varepsilon_{2}\right)r^{d}}, \end{array} \end{array}$$

for $t<T_{\alpha }$ and

(18) $$\begin{array}{c} \begin{array}{ccc} v_{s_{1}}=0, & \;\; & v_{s_{2}}=-\frac{\left(\omega_{\beta }-1\right)\;\dot{\beta }\;\beta^{d}}{r^{d}},\\ v_{f_{1}}=0, & \;\; & v_{f_{2}}=\frac{-\left(\varepsilon_{2}-\varepsilon_{1}\right)\;\dot{\beta }\;\beta^{d}}{\left(1-\varepsilon_{2}\right)r^{d}}, \end{array} \end{array}$$

for $t>T_{\alpha }$ using Equations (14) and (15). For Case 2, applying Equations (15) and (16) gives

(19) $$\begin{array}{c} \begin{array}{ccc} v_{s_{1}}=-\frac{\left(\omega_{\alpha }-1\right)\;\dot{\beta }\;\beta^{d}}{r^{d}}, & \;\; & v_{s_{2}}=-\frac{\left(\omega_{\alpha }\;\omega_{\beta }-1\right)\dot{\beta }\beta^{d}}{r^{d}},\\ v_{f_{1}}=\frac{\dot{\beta }\;\beta^{d}}{r^{d}}, & v_{f_{2}}=\frac{\dot{\beta }\;\beta^{d}}{r^{d}}, \end{array} \end{array}$$

for $t\leq T_{\alpha =\beta }$, and the velocities for $T_{\alpha =\beta }<t<T_{\alpha }$ and $t>T_{\alpha }$ are then Equations (17) and (18), respectively. Using the formulation in Equation (17) for $v_{s_{2}}$ and the boundary condition $\dot{S}=v_{s_{2}}\left(S,t\right)$, we obtain

(20) $$\begin{array}{c} S=S_{0}\;{\left[-\left(\omega_{\beta }-1\right)\;\beta^{d+1}-\omega_{\beta }\left(\omega_{\alpha }-1\right)\;\alpha^{d+1}+\omega_{\alpha }\;\omega_{\beta }\right]}^{1/d+1}; \end{array}$$

this formula is correct for $t<T_{\alpha =\beta }$ in Case 2 on insertion of α=β and in the final phase for both cases on substitution of α=0. From this, we can deduce the final size, $S_{\infty }$, of the magnesium carbonate block on substitution of α=β=0 into Equation (20), giving

(21) $$\begin{array}{c} S_{\infty }=S_{0}\;{\left(\omega_{\alpha }\omega_{\beta }\right)}^{1/(d+1)}; \end{array}$$

this can be calculated a priori from the total volume fraction change from converting Mg to MgCO_3_ being ${\left(S_{\infty }/S_{0}\right)}^{1+d}=\omega_{\alpha }\omega_{\beta }$.

There are no further exact solutions to be obtained and variables $\alpha ,\beta ,W_{1},W_{2}$ and $C_{2}$ need to be resolved numerically from Equations (9), (10) and (11)–(16).

Table 3 shows the data used for each of the parameters other than initial radius $S_{0}$. There are three parameters $\varepsilon_{1},\varepsilon_{2}$ and *k* for which information appears to be limited; these will be investigated further for their effects on the degradation behaviour of magnesium metal and the dynamics of biphasic corrosion layers.

### 2.3. Non-Dimensionalisation

Of the various candidates for a suitable scaling in time, none stand out as providing any particular advantage here, we choose the approximate timescale for which carbon dioxide diffuses across a reference distance $S_{0}^{*}$ (choosing $S_{0}^{*}=1$ cm). We rescale the water and carbon dioxide variables with ambient mass concentrations $W_{0}^{*}$ and $C_{0}^{*}$ and hence write

$$\begin{array}{c} t=\frac{{S_{0}^{*}}^{2}}{D_{c}}\hat{t},\;r=S_{0}^{*}\hat{r},\;W_{1}=W_{0}^{*}\hat{W_{1}},\;W_{2}=W_{0}^{*}\hat{W_{2}},\;C_{2}=C_{0}^{*}\hat{C_{2}},\;\alpha =S_{0}^{*}\hat{\alpha },\;\beta =S_{0}^{*}\hat{\beta },\;S=S_{0}^{*}\hat{S},\; \end{array}$$

and $v_{*}=D_{C}{\hat{v}}_{*}/S_{0}^{*}$, where the quantities with hats are dimensionless. Using the data in Table 3, the scaling implies that $\hat{t}=1$ represents about 14.5 h. Let

(22) $$\begin{array}{c} {\hat{S}}_{0}=\frac{S_{0}}{S_{0}^{*}},\;\;{\hat{D}}_{W}=\frac{D_{W}}{D_{C}},\;\;\gamma_{0}=\frac{M_{W}\rho_{0}}{M_{0}W_{0}^{*}},\;\;\gamma_{1}=\frac{M_{W}\rho_{1}}{M_{1}W_{0}^{*}},\;\;\gamma_{2}=\frac{M_{C}\rho_{1}}{M_{1}C_{0}^{*}},\;\;\kappa =\frac{S_{0}^{*}W_{0}^{*}}{D_{C}}\;k, \end{array}$$

noting that $\omega_{\alpha }$ and $\omega_{\beta }$ are already dimensionless; then, on dropping the hats for clarity, we obtain the system

(23) $$\begin{array}{cc} \frac{\partial \;W_{1}}{\partial \;t}+v_{f_{1}}\;\frac{\partial \;W_{1}}{\partial \;r}-\frac{D_{W}}{r^{d}}\frac{\partial }{\partial \;r}\;\left(r^{d}\frac{\partial \;W_{1}}{\partial \;r}\right)\;\;= & \;\;0, \end{array}$$

(24) $$\begin{array}{cc} \frac{\partial \;W_{2}}{\partial \;t}+v_{f_{2}}\;\frac{\partial \;W_{2}}{\partial \;r}-\frac{D_{W}}{r^{d}}\frac{\partial }{\partial \;r}\;\left(r^{d}\frac{\partial \;W_{2}}{\partial \;r}\right)\;\;= & \;\;0, \end{array}$$

(25) $$\begin{array}{cc} \frac{\partial \;C_{2}}{\partial \;t}+v_{f_{2}}\;\frac{\partial \;C_{2}}{\partial \;r}-\frac{1}{r^{d}}\frac{\partial }{\partial \;r}\;\left(r^{d}\frac{\partial \;C_{2}}{\partial \;r}\right)\;\;= & \;\;0, \end{array}$$

with velocities for each scenario the same as Equations (17)–(19). Initial conditions are

(26) $$\begin{array}{c} \alpha \left(0\right)=\beta \left(0\right)=S\left(0\right)=S_{0}. \end{array}$$

For Case 1, k→∞, we have the following boundary conditions for $t<T_{\alpha }$,

(27) $$\begin{array}{c} \begin{array}{c} W_{1}\left(\alpha ,t\right)=0,\;W_{1}\left(\beta ,t\right)=W_{2}\left(\beta ,t\right),\;W_{2}\left(S,t\right)=1,\;C_{2}\left(\beta ,t\right)=0,\;C_{2}\left(S,t\right)=1, \end{array} \end{array}$$

interface conditions,

(28) $$\begin{array}{c} \begin{array}{cc} r=\alpha (t): & -\left(1-\varepsilon_{1}\right)\;D_{W}\;\partial_{r}W_{1}=2\;\gamma_{0}\;\dot{\alpha },\\ r=\beta (t): & D_{W}\;\left(\left(1-\varepsilon_{1}\right)\;\partial_{r}W_{1}-\left(1-\varepsilon_{2}\right)\;\partial_{r}W_{2}\right)=-\gamma_{1}\left(\dot{\beta }+\frac{\left(\omega_{\alpha }-1\right)\;\alpha^{d}\dot{\alpha }}{\beta^{d}}\right),\\ & -\left(1-\varepsilon_{2}\right)\partial_{r}C_{2}=\gamma_{2}\;\left(\dot{\beta }+\frac{\left(\omega_{\alpha }-1\right)\;\alpha^{d}\dot{\alpha }}{\beta^{d}}\right), \end{array} \end{array}$$

and analytical solution for *S*,

(29) $$\begin{array}{c} S\;=\;S_{0}{\left(\omega_{\alpha }\omega_{\beta }-\left(\omega_{\beta }-1\right)\beta^{d+1}-\omega_{\beta }\left(\omega_{\alpha }-1\right)\alpha^{d+1}\right)}^{1/(d+1),} \end{array}$$

and for $t>T_{\alpha }$,

(30) $$\begin{array}{c} \begin{array}{cc} r=0: & \partial_{r}W_{1}\;=\;0,\\ r=\beta (t): & D_{W}\;\left(\left(1-\varepsilon_{1}\right)\;\partial_{r}W_{1}-\left(1-\varepsilon_{2}\right)\;\partial_{r}W_{2}\right)=-\gamma_{1}\dot{\beta },\;-\left(1-\varepsilon_{2}\right)\partial_{r}C_{2}=\gamma_{2}\;\dot{\beta }, \end{array} \end{array}$$

with analytical solution

(31) $$\begin{array}{c} S=S_{0}{\left(\omega_{\alpha }\omega_{\beta }-\left(\omega_{\beta }-1\right)\beta^{d+1}\right)}^{1/(d+1)}. \end{array}$$

For Case 2 and $t\leq T_{\alpha =\beta }$, we have the following boundary conditions:

(32) $$\begin{array}{c} \begin{array}{c} W_{2}\left(S,t\right)=1,\;C_{2}\left(S,t\right)=1, \end{array} \end{array}$$

with interface conditions,

(33) $$\begin{array}{c} \begin{array}{cc} r=\alpha (t)=\beta (t): & \gamma_{0}\dot{\beta }=-\kappa W_{2}^{2},\;-D_{W}\;\left(1-\varepsilon_{2}\right)\;\partial_{r}W_{2}=\gamma_{1}\;\omega_{\alpha }\;\dot{\beta },\\ & -\left(1-\varepsilon_{2}\right)\partial_{r}C_{2}=\gamma_{2}\;\omega_{\alpha }\;\dot{\beta }, \end{array} \end{array}$$

and analytical solution

(34) $$\begin{array}{c} S\;=\;S_{0}{\left(\beta^{d+1}+\omega_{\alpha }\omega_{\beta }\left(1-\beta^{d+1}\right)\right)}^{1/(d+1)}. \end{array}$$

For $t>T_{\alpha =\beta }$, we have, in addition to Equation (32), the boundary conditions

(35) $$\begin{array}{c} \begin{array}{c} \gamma_{0}\dot{\alpha }=-\kappa W_{1}^{2},\;W_{1}\left(\beta ,t\right)=W_{2}\left(\beta ,t\right),\;C_{2}\left(\beta ,t\right)=0, \end{array} \end{array}$$

whereby, for $T_{\alpha =\beta }<t<T_{\alpha }$, we have Equations (28) and (29) and, for $t>T_{\alpha },$ we impose Equations (30) and (31).

Table 4 displays the values for the dimensionless parameters used in the model simulations in the next section.

## 3. Numerical Method and Results

The spacial domains of the system of PDE Equations (23)–(25) are mapped to the unit interval ρ∈[1,2] using the rescaling outlined in Appendix A; the difficulties from the singularities resulting from α=β=S at t=0 at the start of Phases 1.1 and 2.1 and α=β at $t=T_{\alpha =\beta }$ at the start of Phase 2.2 are discussed in Appendix B.

The system of PDE Equations (A1)–(A3) and the appropriate boundary conditions for each of the phases was solved using the method of lines [33] implemented in MATLAB (R2017a, MathWorks). The domains for Zones 1 and 2 are divided into a uniform mesh, not necessarily using the same number of points, and the spatial derivatives are discretised using central differences; upwind scheme for the advection terms was also implemented but usually ran slower. The stiff ODE solver, *ode15s*, was used for the time stepping process.

From the data values listed in Table 3, there is current uncertainty on appropriate values for $\varepsilon_{1},\varepsilon_{2}$ and κ (though $\varepsilon_{1}=0.6$ and $\varepsilon_{2}=0.4$ was chosen for most simulations). We therefore investigate in Section 3.2–Section 3.5, for the three principle geometries, the effect of these parameters on the model solutions, in particular on the degradation times of the original Mg block and the Mg(OH)_2_ layer. We investigate in Section 3.3 the effect of the initial size of the block, which is, of course, an experimentally controllable parameter. In Section 3.6, the significance of the porous media assumption in the current model is examined. We note that all of the results shown are the dimensionless form of the variables, whereby one space unit represents 1 cm and one time unit represents about 14.5 h.

### 3.1. Magnesium Degradation

**Figure 2:**
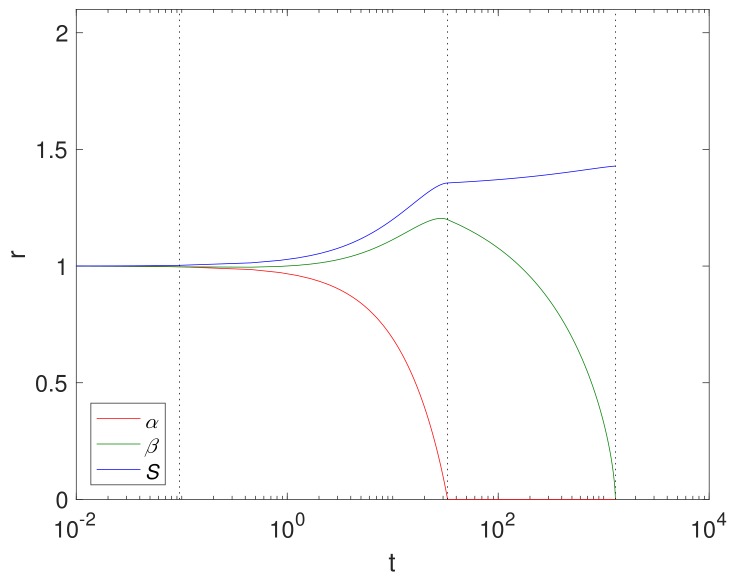
Plots of the dimensionsionless variables α,β and *S* against *t* in cylindrical geometry using $\varepsilon_{1}=0.6$, $\varepsilon_{2}=0.4$, κ=0.04, the parameters in Table 4 and $S_{0}=1$. The dashed lines show $t=T_{\alpha =\beta }$ (**left**), $t=T_{\alpha }$ (**middle**) and $T_{\beta }$ (**right**).

An example simulation using a finite reaction rate κ (Case 2) is shown in Figure 2 using cylindrical geometry, where κ≈0.04, (corresponding to $k=0.07\;{\mathrm{cm}}^{4}/g\cdot s$ in Table 3), $\varepsilon_{1}=0.6$ and $\varepsilon_{2}=0.4$. The size of Mg and its compounds over time are displayed in Figure 2 with the dashed lines showing $T_{\alpha =\beta },T_{\alpha }$ and $T_{\beta }$. We note that the Mg block degrades relatively quickly at t=O(10), whilst the Mg(OH)_2_ takes $t=O(10^{3})$. This is largely due to a relatively low concentration of CO_2_ compared to H_2_O in the fluid phase.

**Figure 3:**
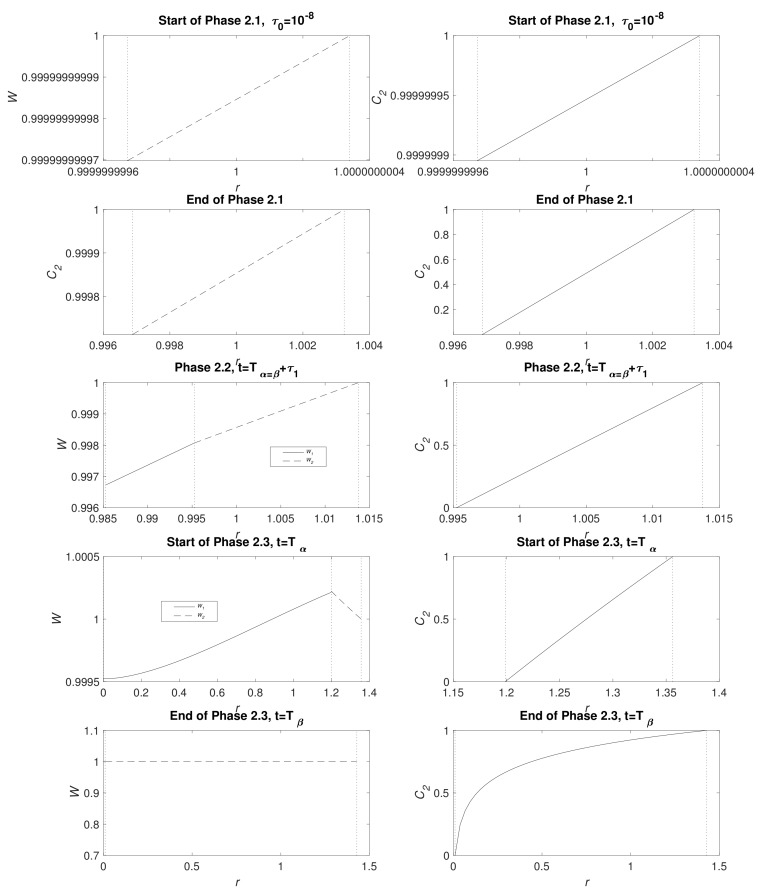
Plots of the dimensionsionless variables concentrations $W_{1},W_{2}$ (left) and $C_{2}$ (right) at, from top to bottom, the start of Phases 2.1 ($t=\tau_{0}=10^{-8}$), end of Phase 2.1 ($t=T_{\alpha =\beta }\approx 0.095$), Phase 2.2 ($t=T_{\alpha =\beta }+\tau_{1}$, with $\tau_{1}=0.356$, see Appendix B.3), start of Phases 2.3 ($t=T_{\alpha }\approx 33.1$) and the end of 2.3 ($t=T_{\beta }\approx 1291$) in cylindrical geometry. In the left-hand panel, the solid lines are $W_{1}$ and the dashed lines $W_{2}$. The vertical dotted lines indicate from right to left, r=S, r=β (top 3 plots) and r=β (top 2 plots). The parameters are $\varepsilon_{1}=0.6$, $\varepsilon_{2}=0.4$, κ≈0.04, $S_{0}=1$ and the rest listed in Table 4.

Figure 3 displays water and CO_2_ concentration distribution at the start of Phases 2.1, end of Phase 2.1 ($t=T_{\alpha =\beta }$), Phase 2.2 at the point the full system is solved numerically (see Appendix B.3), the start of Phase 2.3 ($t=T_{\alpha }$) and the end of Phase 2.3 ($t=T_{\beta }$); the times *t* are detailed in the caption. In a short time, there is only a very narrow MgCO_3_ layer present and, as expected, the water and CO_2_ are very nearly uniform r∈(β,S). Furthermore, CO_2_ is not initially exhausted by the conversion reaction of Mg(OH)_2_ to MgCO_3_ at r=β, but, in time, it descends, reaching zero as Phase 2.2 begins. As time advances, clear gradients in concentrations emerge and, whilst β−α and S−β remain small, the concentrations of water and CO_2_ appear linear. The upward kink in the water distribution is due to production at r=β in the conversion reaction Equation (2), even exceeding the exterior concentration as, locally, water replaces CO_2_ molecules. During Phase 2.3, when there is no more Mg remaining, the water distribution $W_{1}$ tends to a uniform distribution via diffusion and the zero flux condition at r=0. By the end of Phase 2.3, the profiles of $W_{2}$ and $C_{2}$ are no longer linear and CO_2_ concentration forms a boundary layer in the vicinity of r=β.

### 3.2. Effects of Geometry

**Figure 4:**
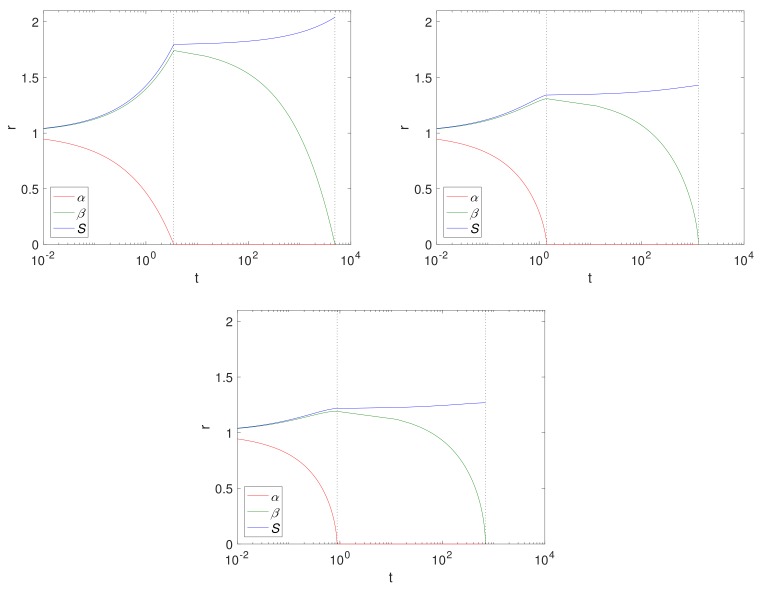
Plots of the dimensionsionless variables α,β and *S* against *t*, from left to right, Cartesian, cylindrical and spherical geometry using $\varepsilon_{1}=0.6$, $\varepsilon_{2}=0.4$, κ→∞, the parameters in Table 4 and $S_{0}=1$. The dashed lines show $t=T_{\alpha }$ (**left**) and $T_{\beta }$ (**right**).

Figure 4 shows α,β and *S* over time for a Case 1 (κ→∞) example, using fixed solid fractions, $\varepsilon_{1}=0.6$ and $\varepsilon_{2}=0.4$ and an initial radius of $S_{0}=1$ (representing 1 cm). The results are displayed left to right for Cartesian, cylindrical and spherical geometries. The dashed lines separate the two-phases, Phase 1.1 and Phase 1.2. Here, the geometry is such that the size of the Mg block is greater in the Cartesian case, thus there is more of it to convert to Mg(OH)_2_ and ultimately degrade into MgCO_3_, as can be seen from the final sizes, where $S_{\infty }^{\left[d\right]}$, (d=0,1,2 for the Cartesian, cylindrical and spherical geometry, respectively), $S_{\infty }^{\left[2\right]}<S_{\infty }^{\left[1\right]}<S_{\infty }^{\left[0\right]}$. Comparing the cylindrical case with that of Figure 2, we observe that, as expected, the magnesium layer disappears much faster in κ→∞ case ($T_{\alpha }\approx 1$) than for κ≈0.04 ($T_{\alpha }\approx 33$); but we note that it does not significantly affect the overall degradation time of Mg(OH)_2_. In reality, the limit κ→∞ is unlikely to be realistic for pure or mostly pure magnesium, and represents a metal of low purity. However, as degradation of Mg(OH)_2_ is independent of κ, there is very little difference between the $t=T_{\beta }$ values.

### 3.3. Effect of Magnesium Block Size

**Figure 5:**
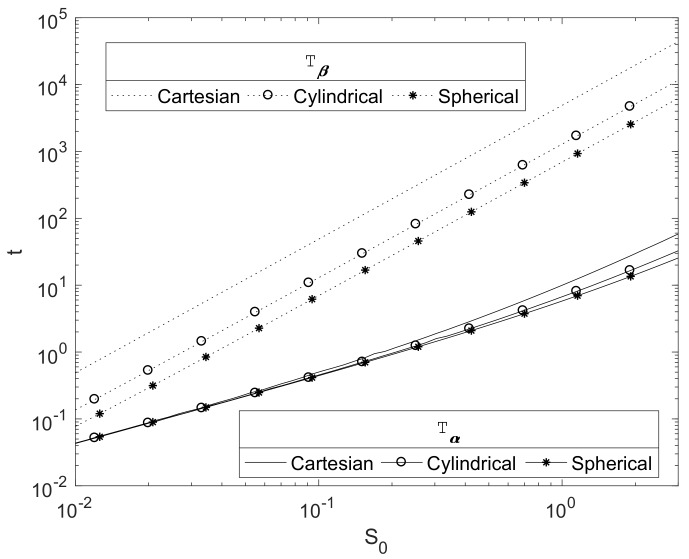
Plots of the dimensionsionless $T_{\alpha }$ and $T_{\beta }$ against the initial magnesium block size, $S_{0}$, for each of the principle geometries. The parameters used are $\varepsilon_{1}=0.6$, $\varepsilon_{2}=0.4$, κ≈0.3 ($k=0.5\;{\mathrm{cm}}^{4}/g\;\mathrm{day}$) and parameters in Table 4.

The scaling presented in Section 2.3 is such that the initial magnesium block size of $S_{0}=1$, used in the simulations up to now, represents 1 cm. Figure 5 plots the relationship between the initial Mg radius and the key degradation timescales $T_{\alpha }$ and $T_{\beta }$ for each of the three principle geometries in a finite κ case. As expected, they show that these timescales increase with the initial radius of the Mg block, $S_{0}$. The plots suggests a power law relationship of $T_{\beta }\propto S_{0}^{2}$. This can be justified due to the concentration of CO_2_ being relatively small, so that $1\ll \gamma_{2}$, and hence the decay of the Mg(OH)_2_ is slow. Here, $\dot{\beta }\ll 1$ and hence $v_{f_{2}}\ll 1$, and then Equation (25) would be expected to tend to the quasi-steady profile, with $\partial_{r}r^{d}\partial_{r}C_{2}∼0$, hence $\partial_{r}C_{2}\left(\beta ,t\right)∼A/\beta^{d}$ for some constant A>0. Writing $r=S_{0}\bar{r}$ and $\beta =S_{0}\bar{\beta }$, then, from Equation (30), we obtain

$$\begin{array}{c} {\frac{d\;\bar{\beta }}{d\;t}}^{\;d+1}\;∼\;-\frac{\left(d+1\right)\left(1-\varepsilon_{2}\right)A}{S_{0}^{2}}, \end{array}$$

thus ${\bar{\beta }}^{d+1}∼\beta_{0}^{d+1}-t\;\left(d+1\right)\left(1-\varepsilon_{2}\right)A/S_{0}^{2}$; hence, we can crudely estimate

(36) $$\begin{array}{c} T_{\beta }\;∼\;S_{0}^{2}\;\frac{B}{\left(1-\varepsilon_{2}\right)}, \end{array}$$

where constant $B=\beta_{0}^{d+1}/A\left(d+1\right)>0$, thus $T_{\beta }\propto S_{0}^{2}$ as shown in Figure 5.

### 3.4. Effect of Porosity of the Mg(OH)_2_ and MgCO_3_ Layers

**Figure 6:**
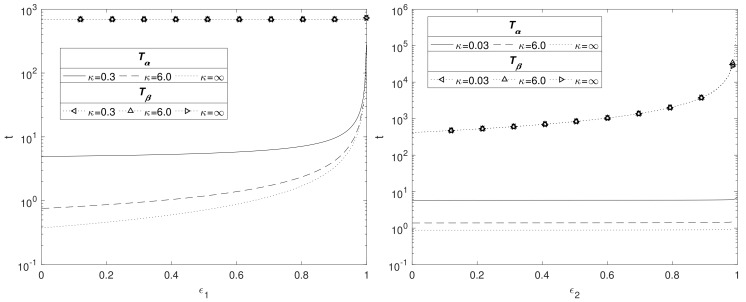
Plots of the dimensionsionless $T_{\alpha }$ and $T_{\beta }$ against $\varepsilon_{1}$ (**left**) and $\varepsilon_{2}$ (**right**) for κ=0.03,6 and κ→∞, with the remaining parameters listed in Table 4 and $S_{0}=1$.

In Figure 6, the effect of $\varepsilon_{1}$ (left) and $\varepsilon_{2}$ (right) on the time scales $T_{\alpha }$ and $T_{\beta }$ is shown for κ=0.3,6 and κ→∞. The left side of Figure 6 shows that $T_{\alpha }$ increases with the solid fraction, rising sharply as $\varepsilon_{1}\to 1$. This is to be expected because as $\varepsilon_{1}$ increases there is less space for water to flow through the Mg(OH)_2_ layer, hence decreasing the rate at which water reaches the Mg interface. However, the solid fraction does not have an impact on the degradation time for Mg(OH)_2_ as the transport of CO_2_ in the MgCO_3_ layer governs this process. The figure emphasises the importance of the hydroxide layer at slowing the degradation of the metal core by impeding the passage of water. The nonlinear relationship as predicted by the current model is in accordance with experimental data [34], whilst decreasing in porosity having the effect of enhancing longevity is consistent with Sun et al. [35].

The right side of Figure 6 shows the effects of changing $\varepsilon_{2}$ whilst keeping $\varepsilon_{1}$ fixed at 0.6. The solid fraction of MgCO_3_ does not appear to have an effect on $T_{\alpha }$, but does affect $T_{\beta }$. Here, for $t<T_{\alpha }$, the thickness of the MgCO_3_ layer, S−β, is fairly small and appears not to be sufficient to impede significantly the passage of water across it, thus $T_{\alpha }$ remains approximately constant. Although, $T_{\alpha }$ varies between the κ values, it is small compared to $T_{\beta }$, and the conversion of Mg(OH)_2_ to MgCO_3_ being independent of κ means the plots are superimposed. As $\varepsilon_{2}$ increases, it is CO_2_ that is impeded by the smaller void fraction, leading to the sharp rise in time $T_{\beta }$ as $\varepsilon_{2}\to 1$. Using the argument in Section 3.3 in formulating Equation (36) for large $\gamma_{2}$, we then expect $T_{\beta }\propto 1/\left(1-\varepsilon_{2}\right)$; this relationship matches the numerics very well, suggesting that $T_{\beta }\to \infty$ as $\varepsilon_{2}\to 1^{-}$.

### 3.5. Effect of Rate of Reaction at Magnesium Interface

**Figure 7:**
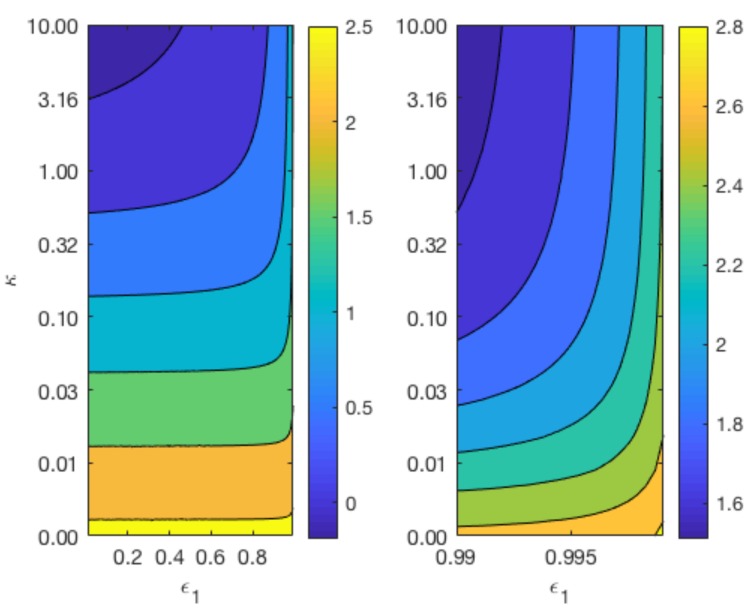

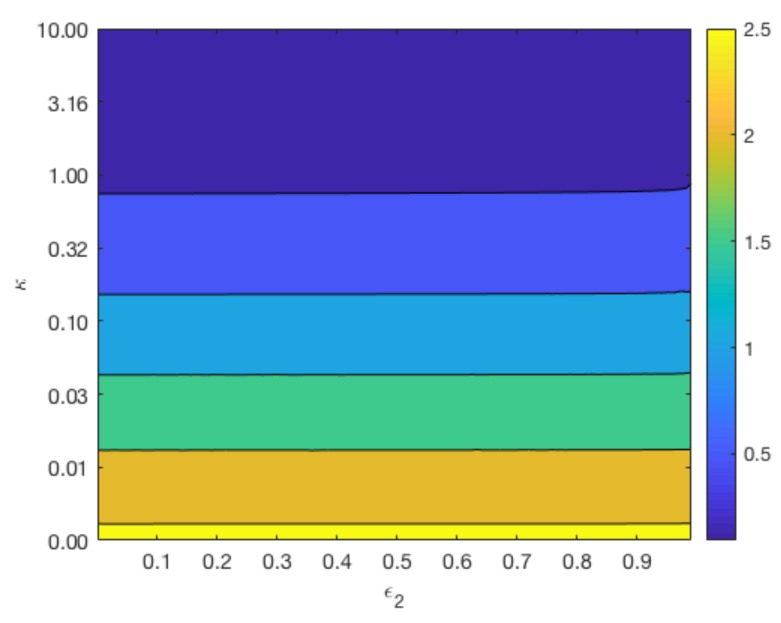
Contour map of the dimensionsionless $T_{\alpha }$ for κ with $\varepsilon_{1}=0.4$ (**top**) and κ against $\varepsilon_{2}$ with $\varepsilon_{1}=0.6$ (**bottom**) in spherical geometry. The remaining parameters are listed in Table 4 and $S_{0}=1$.

Figure 7 displays contours of $T_{\alpha }$ for a range of values for κ and $\varepsilon_{1}$ whilst keeping $\varepsilon_{2}$ constant at 0.4 (top plots), and a range of values for κ and $\varepsilon_{2}$ whilst keeping $\varepsilon_{1}$ constant at 0.6 (bottom plot). The variation in the parameter κ reflects the different degradation rates across various magnesium alloys. As can be observed from the top plot of Figure 7, the longevity of Mg increases as κ and the void fraction $1-\varepsilon_{1}$ decreases. In agreement with Figure 6, the lower plot shows that changes in $\varepsilon_{2}$ do not have a significant impact on the degradation time of Mg, but a smaller κ lengthens the degradation time of Mg.

**Figure 8:**
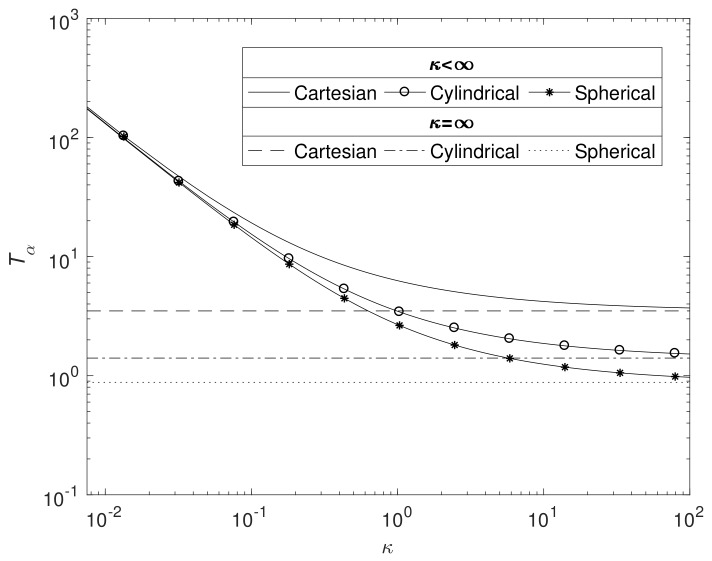
Plots of the dimensionsionless $T_{\alpha }$ against κ for the three principle geometries, with $\varepsilon_{1}=0.6$$\;\varepsilon_{2}=0.4$, $S_{0}=1$ and the parameters listed in Table 4.

Figure 8 displays the effects on $T_{\alpha }$ as κ changes in all three geometries ($\varepsilon_{1}=0.6$ and $\varepsilon_{2}=0.4$). As expected, the time for the total degradation of pure magnesium increases as the reaction rate diminishes, whilst the curves tend to the Case 1 solutions as κ→∞. For the reasons outlined in Section 3.2, Mg takes the longest time to degrade in Cartesian, then cylindrical and then spherical geometry, though the differences are less noticeable on a logged axis as κ→0.

### 3.6. Role of Advection

**Figure 9:**
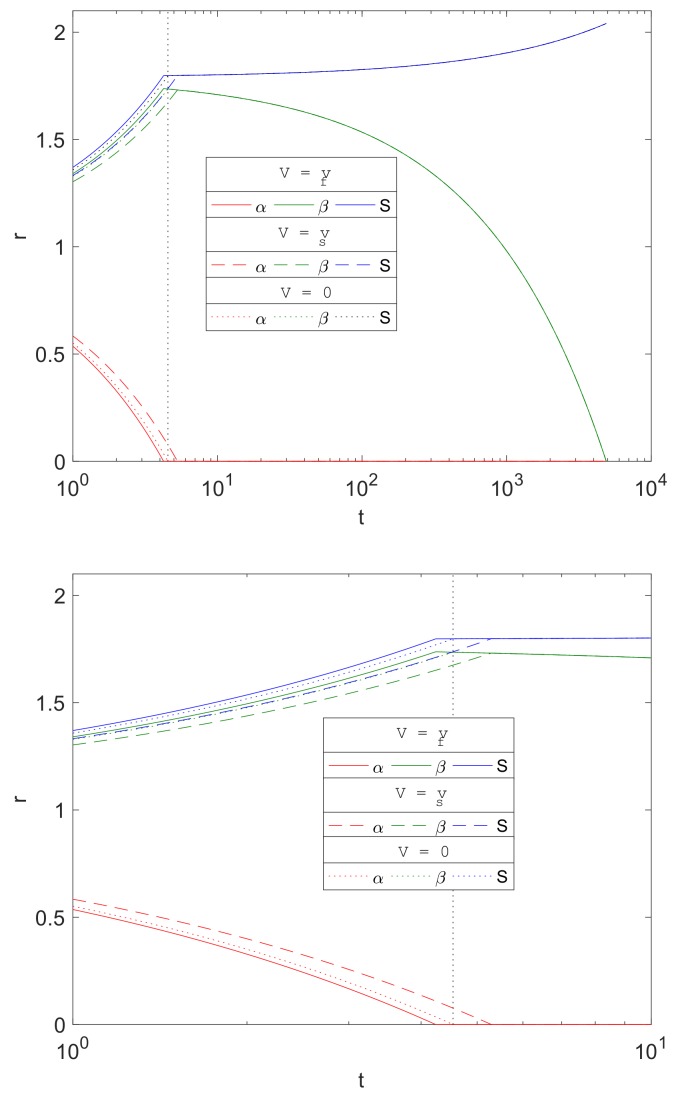
Plots of the evolution of the dimensionsionless variables α,β and *S* resulting from three choices of fluid phase advection velocity, $V_{i}$, namely $V_{i}=v_{f_{i}}$ (as proposed in the current model, solid lines), $V_{i}=0$ (dotted) and $V_{i}=v_{s_{i}}$ (as used in [16], dashed). The left plot shows the full evolution of the interfaces and the right plot shows the results around $t=T_{\alpha }$. The results are using cylindrical geometry, with $\varepsilon_{1}=0.6$, $\varepsilon_{2}=0.4$ and parameters in Table 4 and $S_{0}=1$.

The porous media assumption of the corrosion by-products is a novel feature of the current work in metal corrosion studies. The formation of Mg(OH)_2_ and MgCO_3_ crystal structures allows transport of water and carbon dioxide through its pores. The separate treatment of the resulting fluid and solid phase velocities is in contrast to [16], in which they assumed that the transport of the diffusive species is supplemented by that of the solid phase motion; this presumably reflects these molecules being somehow connected to and dragged along by the crystal structure. Figure 9 compares the evolution of α,β and *S* from three choices of advective flux velocities, $V_{i}$, of water and CO_2_, namely

Case (i). The current model based on porous media assumption ($V_{i}=v_{f_{i}}$, solid lines).
Case (ii). Zero advective transport ($V_{i}=0$, dotted lines), i.e., $v_{f_{i}}$ set to zero in Equations (23)–(25) and in the boundary conditions.
Case (iii). Advective transport equal to the solid phase velocity, as in [16] ($V_{i}=v_{s_{i}}$, dashed lines), i.e., $v_{f_{i}}$ swapped with $v_{s_{i}}$ in Equations (23)–(25) and in the boundary conditions.

The plots show that there is little difference qualitatively with the results between the cases, and the only visible difference being in the stages up to about $t=T_{\alpha }$. Mg is predicted to degrade slightly faster using the current model’s assumptions ($V_{i}=v_{f_{i}}$) than the zero advection case ($V_{i}=0$) and, in turn, is predicted to be faster than that using $V_{i}=v_{s_{i}}$ as the advection flux. This is due to the signs of the advective fluxes being $v_{f_{i}}<0<v_{s_{i}}$, as the reactions generates local solid volume increases, since $w_{\alpha },w_{\beta }>1$. Thus, negative advective flux in case (i) implies that there is a background inward drift of the reactants towards the reaction sites and hence the overall corrosion rates will be predicted to be faster than of case (ii), with zero drift, and case (iii), where the reactants are drawn away by the drift. For $t>T_{\alpha }$, the differences are maintained, but, from the figure, the choice of advective flux appears to have little effect on β over a longer period of time.

## 4. Discussion

In this paper, a partial-differential equation system, with moving boundaries and interfaces, was used to describe the degradation of a magnesium block in aqueous media. This is a first step of modelling degradation of Mg or Mg alloy based orthopaedic implants in biologically relevant environments. The model considers the diffusion and advection of reactants through porous media in the crystal structures generated by reactions with magnesium and its products. Novel features in terms of metal corrosion modelling is the consideration of porous media flow in the crystal structures and the explicit consideration of MgCO_3_. The model was analysed numerically, but small time asymptotic solutions were needed to deal with singularities at initial and certain time points. In principle, the modelling approach is generic and can be used or adapted to model the corrosive process of any metals or alloys.

The porous media assumption leads to the explicit consideration of the solid phase flow, through manufacturing of crystals at the reaction interfaces, and a fluid phase flow to avoid a vacuum. In 1D, using the classical Cartesian, cylindrical or spherical geometries, a closed system of equations can be derived using mass conservation alone. The flow velocities are analytically solvable, and the advective flow of the reactants in the fluid phase does not complicate the model significantly, from one that assumes diffusion as the only means of reactant transport. The investigation in Section 3.6 compared results using three different advection assumptions, whereby $V_{i}=v_{f_{i}}$ (current model) and $V_{i}=v_{s_{i}}$ (used in [16]), representing extreme cases of physically relevant advective velocity $V_{i}$. The results showed that the choice of advection term can notably affect the predicted time of pure magnesium degradation, though, in the long term, there is little difference in the predicted results. The results of Section 3.2 show that geometry has a significant effect on timescales for degradation and this can impact the shapes of materials used in an implant. For more accurate predictions of corrosion of complex shapes, the problem needs to be studied in two or three dimensions, as has been considered using different modelling approaches in, for example, [22,35]. Extending the current modelling approach to consider 2D or 3D, non-simple geometries poses non-trivial modelling challenges. The increase in number of variables will require constitutive assumptions on the mechanical properties of the Mg(OH)_2_ and MgCO_3_ layers to close the system [36].

The model consists of three parameters *k*, $\varepsilon_{1}$ and $\varepsilon_{2}$ that are not readily available from the literature. The results of Section 3.4 and Section 3.5 show that these can be tuned to predict a wide range of results in terms of timescales for the vanishing of pure magnesium, $t=T_{\alpha }$, and Mg(OH)_2_ layer, $t=T_{\beta }$. However, there is scope for these parameters to be estimated based on appropriate in vitro data. For example, data from time-course measurements of the proportion of constituents of small, spherical magnesium or magnesium alloy beads, immersed in appropriate media. The use of small beads, say around 0.1–1 mm radius (see Section 3.3), should ensure the experiment to be completed in a practicable and cheap time frame, whilst them being spherical enables direct application of the model to calibrate *k*, $\varepsilon_{1}$ and $\varepsilon_{2}$ with the data. The interpretation of *k* can be extended to the corrosion rate of different quality of magnesium metal and its alloy. In particular, the presence of impurity and the grain size of micro-structures, depending on the preparation methods used, can have dramatic effects on the corrosion rate together with environmental factors [37,38,39,40]. A further experiment involves the use of computed tomography (CT) images, which enables spatial details of the macroscale crystal structure that can be used to obtain direct measurement of $\varepsilon_{1}$ and $\varepsilon_{2}$. The model, with these tuned or determined parameters, provides a starting point to predict the corrosion properties of much larger magnesium pellets and in any 2D or 3D extension outlined above.

The current model describes the corrosion of a smooth Mg block in an aqueous media. The constituents of the media that an orthopaedic implant will be exposed to is more complex and may have significant effects on the corrosion behaviour. For example, lower pH corrodes the Mg(OH)_2_ and MgCO_3_, so that the pure Mg is more exposed to the environment and accelerating its corrosion [11,41]. Furthermore, the tougher outer layer of MgCO_3_ will itself be corroded and the resulting magnesium ions will eventually disperse and be excreted by the host; the modelling of this corrosion process leads to a modified boundary condition on r=S. Chloride ions in plasma will also react with Mg(OH)_2_ to form MgCl_2_ [9]; here, the model can be extended to consider two reactive species for Mg(OH)_2_, and assume, for simplicity, that the outer layer consists of an isotropic mixture of MgCO_3_ and MgCl_2_. In practice, the magnesium block will be pitted and have holes that will presumably affect its corrosive properties as well [42]; this is currently being explored by the authors. There is thus plenty of scope to improve the current model, in order to describe more realistically the corrosion properties of magnesium based orthopaedic implants in vivo. Nevertheless, the current model provides a promising initial step into a theoretical understanding of magnesium corrosion, hopefully providing useful insights to help make informed decisions on the experimental direction and design of magnesium based implant materials.

## 5. Conclusions

To conclude, we summarise the key points from the preceding sections.

- Mathematically modelled Mg (or Mg alloy) corrosion in aqueous environments (e.g., cell culture medium) and the corrosion products Mg(OH)_2_ and MgCO_3_, forming up to three discrete regions of for Mg, Mg(OH)_2_ and MgCO_3_, the boundaries of which move in time.
- The corrosion products are treated as porous media, whereby fluid (water, CO_2_) and solid (Mg and its compounds) phases move separately. The reactants are transported via diffusion and advection.
- The model is an advection–diffusion system with multiple moving boundaries, marking the coordinate of the interfaces between the solid phase species.
- Many of the parameters are obtainable from literature, leaving three free parameters: the reaction rate between Mg (or Mg alloy) and water (*k*), and the solid volume fractions of Mg(OH)_2_ ($\varepsilon_{1}$) and MgCO_3_ ($\varepsilon_{2}$) layers.
- There are two key timescales for Mg corrosion process, namely that of complete corrosion of the original Mg block ($T_{\alpha }$) and Mg(OH)_2_ ($T_{\beta }$), so that at $T_{\beta }$ all that remains is a MgCO_3_ block. Numerical solutions demonstrated that, over a wide range of parameters,
  – $T_{\alpha }\ll T_{\beta }$, the original Mg block is short-lived relative to the complete corrosion process.
  – *k* and $\varepsilon_{1}$ affect $T_{\alpha }$, whilst having a little effect on $T_{\beta }$. The latter is affected most by $\varepsilon_{2}$. To substantially prolong Mg presence, Mg alloys must have the effect of reducing the reaction rate *k*.
  – geometry has an impact on the corrosion timescales.
  – complete corrosion is described well by the law $T_{\beta }\propto S_{0}^{2}/\left(1-\varepsilon_{2}\right)$, where $S_{0}$ is the original radius (or size) of the Mg block.
- Relatively simple in vitro experimentation and CT scans can be used to inform the free parameters, thereby enabling the model to predict the outcome in situations more challenging to undertake experimentally.

## Figures and Tables

**Table 1:** Notation used in the model, where $\rho_{i}=M_{i}\mu_{i},\omega_{\alpha }=\mu_{0}/\mu_{1}$ and $\omega_{\beta }=\mu_{1}/\mu_{2}$.

Name	Notation
Zone	*i*
Solid fraction	$\varepsilon_{i}$
Mass density	$\rho_{i}$
Mass/Mol	$M_{i}$
Mol/Volume	$\mu_{i}$
Solid velocity	$v_{s_{i}}$
Fluid velocity	$v_{f_{i}}$

**Table 2:** Relevant boundary conditions for each of the Mg corrosion phases for the two cases k→∞ and k<∞.

Phase		Time	b.c.s	$v_{s_{i}}$ and $v_{f_{i}}$
1.1	k→∞	$0\leq t\leq T_{\alpha }$	(13), (15)	(17)
1.2		$T_{\alpha }<t\leq T_{\beta }$	(14), (15)	(17)
2.1	k<∞	$0\leq t\leq T_{\alpha =\beta }$	(16)	(19)
2.2		$T_{\alpha =\beta }<t\leq T_{\alpha }$	(13), (15)	(17)
2.3		$T_{\alpha }<t\leq T_{\beta }$	(14), (15)	(18)

**Table 3:** List of model variables, their interpretation and, where possible, estimated values from the literature. † unknown parameters in the model. “S” indicates standard textbook references and “D” derived from formula in Table 1.

Parameter	Value	Units	Description	Source
$D_{W}$	2.85	${\mathrm{cm}}^{2}/\mathrm{day}$	Diffusion coefficient of H_2_O	[29]
$D_{C}$	1.66	${\mathrm{cm}}^{2}/\mathrm{day}$	Diffusion coefficient of CO_2_	[30]
$M_{0}$	24.3	g/mol	Molecular mass of Mg	S
$M_{1}$	58.3	g/mol	Molecular mass of Mg(OH)_2_	S
$M_{2}$	84.3	g/mol	Molecular mass of MgCO_3_	S
$M_{W}$	18	g/mol	Molecular mass of H_2_O	S
$M_{C}$	44	g/mol	Molecular mass of CO_2_	S
$\rho_{0}$	1.74	$g/{\mathrm{cm}}^{3}$	Mass density of Mg	S and [31]
$\rho_{1}$	2.34	$g/{\mathrm{cm}}^{3}$	Mass density of Mg(OH)_2_	S
$\rho_{2}$	2.96	$g/{\mathrm{cm}}^{3}$	Mass density of MgCO_3_	S
$W_{0}^{*}$	1	$g/{\mathrm{cm}}^{3}$	Concentration of H_2_O in human body	S
$C_{0}^{*}$	0.0011	$g/{\mathrm{cm}}^{3}$	Concentration of CO_2_ in human body	[32]
$w_{\alpha }$	1.8	-	Molar density ratio of Mg and Mg(OH)_2_	D
$w_{\beta }$	1.1	-	Molar density ratio of Mg(OH)_2_ and MgCO_3_	D
$\varepsilon_{1}$	†	-	Fraction of magnesium hydroxide	-
$\varepsilon_{2}$	†	-	Fraction of magnesium carbonate	-
*k*	†	${\mathrm{cm}}^{4}/g\;\mathrm{day}$	Rate of reaction between Mg and H_2_O	-

**Table 4:** List of dimensionless parameter values calculated from the values listed in Table 3 and Equation (22); † being the free parameters.

Parameter	Value
$D_{W}$	1.5625
$\gamma_{0}$	1.2889
$\gamma_{1}$	0.7225
$\gamma_{2}$	1605.5
$w_{\alpha }$	1.8
$w_{\beta }$	1.1
$\varepsilon_{1}$	†
$\varepsilon_{2}$	†
κ	†

## Appendix A. Change of Variables

For numerical convenience, the two zones—the hydroxide layer, r∈(α,β) (zone 1), and the carbonate layer, r∈(β,S) (zone 2)—are each mapped to an interval of unit size using the transformation (r,t)→(ρ,τ), namely

$$\begin{array}{cc} r=\alpha +(\beta -\alpha )(\rho -1), & \;\;\;\;r\in [\alpha ,\beta ]⟶\rho \in [1,2],\\ r=\beta +(S-\beta )(\rho -1), & \;\;\;\;r\in [\beta ,S]⟶\rho \in [1,2],\\ t=\tau . \end{array}$$

Substitution into Equations (23)–(24) yields

(A1) $$\begin{array}{c} \frac{\partial \;W_{1}}{\partial \;\tau }+\frac{1}{\beta -\alpha }\left(G_{1}\left(\rho ,\tau \right)+v_{f_{1}}\right)\;\frac{\partial \;W_{1}}{\partial \;\rho }-\frac{D_{W}}{{\left(\beta -\alpha \right)}^{2}}\left[\frac{d(\beta -\alpha )}{\alpha +(\beta -\alpha )(\rho -1)}\;\frac{\partial \;W_{1}}{\partial \;\rho }+\frac{\partial^{2}\;W_{1}}{\partial \;\rho^{2}}\right]=0, \end{array}$$

(A2) $$\begin{array}{c} \frac{\partial \;W_{2}}{\partial \;\tau }+\frac{1}{S-\beta }\left(G_{2}\left(\rho ,\tau \right)+v_{f_{2}}\right)\frac{\partial \;W_{2}}{\partial \;\rho }-\frac{D_{W}}{{\left(S-\beta \right)}^{2}}\left[\frac{d(S-\beta )}{\beta +(S-\beta )(\rho -1)}\;\frac{\partial \;W_{2}}{\partial \;\rho }+\frac{\partial^{2}\;W_{2}}{\partial \;\rho^{2}}\right]=0, \end{array}$$

(A3) $$\begin{array}{c} \frac{\partial \;C_{2}}{\partial \;\tau }+\frac{1}{S-\beta }\left(G_{2}\left(\rho ,\tau \right)+v_{f_{2}}\right)\;\frac{\partial \;C_{2}}{\partial \;\rho }-\frac{1}{{\left(S-\beta \right)}^{2}}\left[\frac{d(S-\beta )}{\beta +(S-\beta )(\rho -1)}\;\frac{\partial \;C_{2}}{\partial \;\rho }+\frac{\partial^{2}\;C_{2}}{\partial \;\rho^{2}}\right]=0, \end{array}$$

where

$$G_{1}\left(\rho ,\tau \right)=\left(\dot{\alpha }-\dot{\beta }\right)\;\left(\rho -1\right)-\dot{\alpha },\;G_{2}\left(\rho ,\tau \right)=\left(\dot{\beta }-\dot{S}\right)\;\left(\rho -1\right)-\dot{\beta },$$

using the “dot notation” to denote the derivative with respect to τ. For Phases 1.1 and 2.2, the fluid phase velocities are

$$v_{f_{1}}=\frac{\dot{\alpha }\;\alpha^{d}}{{\left[\alpha +(\beta -\alpha )(\rho -1)\right]}^{d}},\;v_{f_{2}}=\frac{\left(1-\varepsilon_{1}\right)\;\dot{\alpha }\;\alpha^{d}-\left(\varepsilon_{2}-\varepsilon_{1}\right)\;\dot{\beta }\;\beta^{d}}{\left(1-\varepsilon_{2}\right){\left[\beta +(S-\beta )(\rho -1)\right]}^{d}},$$

for Phase 2.1,

$$v_{f_{1}}=\frac{\dot{\beta }\;\beta^{d}}{{\left[\alpha +(\beta -\alpha )(\rho -1)\right]}^{d}},\;v_{f_{2}}=\frac{\dot{\beta }\;\beta^{d}}{{\left[\beta +(S-\beta )(\rho -1)\right]}^{d}},$$

and for Phases 1.2 and 2.3,

$$v_{f_{1}}=0,\;v_{f_{2}}=\frac{\left(\varepsilon_{1}-\varepsilon_{2}\right)\;\dot{\beta }\;\beta^{d}}{\left(1-\varepsilon_{2}\right){\left[\beta +(S-\beta )(\rho -1)\right]}^{d}}.$$

The initial conditions are

$$\begin{array}{c} \begin{array}{cc} \tau =0: & \alpha \;=\;\beta \;=\;S\;=\;S_{0}. \end{array} \end{array}$$

For all phases, we have

(A4) $$\begin{array}{c} W_{2}\left(1,\tau \right)=1,\;C_{2}\left(2,\tau \right)=1. \end{array}$$

For Case 1, κ→∞, the conditions for $0<\tau <T_{\alpha }$ are

(A5) $$\begin{array}{cc} & W_{1}\left(1,\tau \right)=0, \end{array}$$

(A6) $$\begin{array}{cc} & W_{1}\left(2,\tau \right)=W_{2}\left(1,\tau \right), \end{array}$$

(A7) $$\begin{array}{cc} & C_{2}\left(1,\tau \right)=0, \end{array}$$

and

(A8) $$\begin{array}{c} \begin{array}{c} -\left(1-\varepsilon_{1}\right)\;\frac{D_{W}}{\beta -\alpha }\;\partial_{\rho }W_{1}\left(1,\tau \right)=2\;\gamma_{0}\;\dot{\alpha },\\ D_{W}\;\left(\frac{1-\varepsilon_{2}}{S-\beta }\;\partial_{\rho }W_{2}\left(1,\tau \right)-\frac{1-\varepsilon_{1}}{\beta -\alpha }\;\partial_{\rho }W_{1}\left(2,\tau \right)\right)=\gamma_{1}\left(\dot{\beta }+\frac{\left(\omega_{\alpha }-1\right)\;\alpha^{d}\dot{\alpha }}{\beta^{d}}\right),\\ -\frac{\left(1-\varepsilon_{2}\right)}{S-\beta }\partial_{\rho }C_{2}\left(1,\tau \right)=\gamma_{2}\;\left(\dot{\beta }+\frac{\left(\omega_{\alpha }-1\right)\;\alpha^{d}\dot{\alpha }}{\beta^{d}}\right), \end{array} \end{array}$$

and, for $\tau >T_{\alpha }$, we impose Equations (A6) and (A7) and

(A9) $$\begin{array}{c} \partial_{\rho }W_{1}\left(1,\tau \right)=0,\;D_{W}\left(\frac{\left(1-\varepsilon_{2}\right)}{S-\beta }\;\partial_{\rho }W_{2}\left(1,\tau \right)-\;\frac{\left(1-\varepsilon_{1}\right)}{\beta -\alpha }\;\partial_{\rho }W_{1}\left(2,\tau \right)\right)=\gamma_{1}\dot{\beta },\\ -\frac{\left(1-\varepsilon_{2}\right)}{S-\beta }\partial_{\rho }C_{2}\left(1,\tau \right)=\gamma_{2}\;\dot{\beta }. \end{array}$$

For Case 2, κ<∞; then, for $0<\tau <T_{\alpha =\beta },$ we impose

(A10) $$\begin{array}{c} \begin{array}{c} \gamma_{0}\;\dot{\beta }=-\kappa \;W_{2}{\left(1,\tau \right)}^{2},\;-D_{W}\;\frac{\left(1-\varepsilon_{2}\right)}{S-\beta }\;\partial_{\rho }W_{2}\left(1,\tau \right)=\gamma_{1}\;\omega_{\alpha }\;\dot{\beta },\\ -\frac{\left(1-\varepsilon_{2}\right)}{S-\beta }\partial_{\rho }C_{2}\left(1,\tau \right)=\gamma_{2}\;\omega_{\alpha }\;\dot{\beta }, \end{array} \end{array}$$

noting α=β here; for $T_{\alpha =\beta }<\tau <T_{\alpha }$, conditions Equations (A6)–(A8) are imposed along with

(A11) $$\begin{array}{c} \dot{\alpha }=-\frac{\kappa \;W_{2}^{2}}{\gamma_{0}}, \end{array}$$

and, for $\tau >T_{\alpha }$, Equations (A6), (A7) and (A9) are imposed.

The spatial mapping is valid for 0≤α<β<S and gives rise to a singularity at τ=0 (where α=β=S) and for Case 2 at $\tau =T_{\alpha =\beta }$ (where α=β). We evade this problem using the small time asymptotic expansion presented in Appendix B.

## Appendix B. Small Time Asymptotics

To handle the singularities at τ=0 and $\tau =T_{\alpha =\beta }$ for Case 2 in the numerical solution, we obtain small time asymptotic solutions of the variables, and use these to provide approximate initial conditions at a small time increment after the singular time points. The relevant phases for this analysis are Phase 1.1, 2.1 and 2.2 and these are discussed separately.

### Appendix B.1. Phase 1.1

Phase 1.1 considers Case 1 when $\tau <T_{\alpha }$. From Equation (A8), we have

(A12) $$\begin{array}{c} \dot{\alpha }=-\left(1-\varepsilon_{1}\right)\frac{D_{W}}{2\gamma_{0}\left(\beta -\alpha \right)}\frac{\partial \;W_{1}}{\partial \;\rho }, \end{array}$$

where β−α≪1 for τ≪1, and then

$$\dot{\alpha }=O\left(\frac{1}{\beta -\alpha }\right),$$

assuming $\frac{\partial \;W_{1}}{\partial \;\rho }=O\left(1\right)$. We start by seeking expansions of the form

$$\begin{array}{c} \alpha ∼S_{0}+a_{1}\tau^{\sigma },\;\beta ∼S_{0}+b_{1}\tau^{\sigma }, \end{array}$$

where $a_{1},b_{1}=O\left(1\right)$ and σ is to be determined. We have $\beta -\alpha =\tau^{\sigma }\left(b_{1}-a_{1}\right)$ and $\dot{\alpha }=\sigma \tau^{\sigma -1}a_{1}$, so, from Equation (A12), it follows that

$$\begin{array}{c} \tau^{2\sigma -1}=1\Rightarrow \sigma =\frac{1}{2}. \end{array}$$

Consequently, the following small time approximations are applied:

(A13) $$\begin{array}{c} \alpha \left(\tau \right)∼S_{0}+a_{1}\;\tau^{1/2},\;\beta \left(\tau \right)∼S_{0}+b_{1}\;\tau^{1/2},\;S\left(\tau \right)∼S_{0}+s_{1}\;\tau^{1/2}. \end{array}$$

As τ→0, we expect $a_{1}<0$, as some Mg will be used up and $s_{1}>b_{1}>0$ as volume is gained from Mg corrosion. Substituting these into the model Equations (A1)–(A3), we obtain at leading order

(A14) $$\begin{array}{cc} \frac{1}{2}\left(\frac{\left(a_{1}-b_{1}\right)\left(\rho -1\right)-a_{1}+a_{1}\;S_{0}^{d}}{b_{1}-a_{1}}\right)\frac{\partial \;W_{1}}{\partial \;\rho }-\frac{D_{W}}{{\left(b_{1}-a_{1}\right)}^{2}}\frac{\partial^{2}\;W_{1}}{\partial \;\rho^{2}}\; & ∼\;0, \end{array}$$

(A15) $$\begin{array}{cc} \frac{1}{2}\left(\frac{\left(b_{1}-s_{1}\right)\left(\rho -1\right)-b_{1}}{s_{1}-b_{1}}+\frac{\left(1-\varepsilon_{1}\right)a_{1}\;S_{0}^{d}-\left(\varepsilon_{2}-\varepsilon_{1}\right)b_{1}\;S_{0}^{d}}{\left(1-\varepsilon_{2}\right)\left(s_{1}-b_{1}\right)}\right)\frac{\partial \;W_{2}}{\partial \;\rho }-\frac{D_{W}}{{\left(s_{1}-b_{1}\right)}^{2}}\frac{\partial^{2}\;W_{2}}{\partial \;\rho^{2}}\; & ∼\;0, \end{array}$$

(A16) $$\begin{array}{cc} \frac{1}{2}\left(\frac{\left(b_{1}-s_{1}\right)\left(\rho -1\right)-b_{1}}{s_{1}-b_{1}}+\frac{\left(1-\varepsilon_{1}\right)a_{1}\;S_{0}^{d}-\left(\varepsilon_{2}-\varepsilon_{1}\right)b_{1}\;S_{0}^{d}}{\left(1-\varepsilon_{2}\right)\left(s_{1}-b_{1}\right)}\right)\frac{\partial \;C_{2}}{\partial \;\rho }-\frac{1}{{\left(s_{1}-b_{1}\right)}^{2}}\frac{\partial^{2}\;C_{2}}{\partial \;\rho^{2}}\; & ∼\;0. \end{array}$$

As τ→0, with interface conditions

(A17) $$\begin{array}{cc} a_{1}\gamma_{0}\left(b_{1}-a_{1}\right)+\left(1-\varepsilon_{1}\right)D_{W}\;\partial_{\rho }W_{1}\left(1,\tau \right)\; & ∼\;0, \end{array}$$

(A18) $$\begin{array}{cc} 2D_{W}\left(\frac{1-\varepsilon_{1}}{b_{1}-a_{1}}\;\partial_{\rho }W_{1}\left(2,\tau \right)-\frac{1-\varepsilon_{2}}{s_{1}-b_{1}}\;\partial_{\rho }W_{2}\left(1,\tau \right)\right)+\gamma_{1}\;\left(b_{1}+a_{1}\left(\omega_{\alpha }-1\right)\right)\; & ∼\;0, \end{array}$$

(A19) $$\begin{array}{cc} b_{1}+\frac{2\;(1-\varepsilon_{2})}{\gamma_{2}\;\left(s_{1}-b_{1}\right)}\partial_{\rho }C_{2}\left(1,\tau \right)+a_{1}\left(\omega_{\alpha }-1\right)\; & ∼\;0, \end{array}$$

and from the analytical solution for *S*,

(A20) $$\begin{array}{c} s_{1}∼-\left[\left(\omega_{\beta }-1\right)b_{1}\;S_{0}^{d}+\omega_{\beta }\left(\omega_{\alpha }-1\right)a_{1}\;S_{0}^{d}\right]. \end{array}$$

By applying the boundary conditions in Equation (A8) and using $W_{1}\left(2\right)=W_{2}\left(1\right)$, Equations (A14)–(A16) have the analytical solution

(A21) $$\begin{array}{c} \begin{array}{c} W_{1}\left(\rho ,t\right)=\frac{W\;(\lambda_{1}-\lambda_{2})}{\lambda_{1}-\lambda_{3}},\\ W_{2}\left(\rho ,t\right)=\frac{\left(\lambda_{4}-\lambda_{5}\right)\;W+\lambda_{5}-\lambda_{6}}{-\lambda_{6}+\lambda_{4}},\\ C_{2}\left(\rho ,t\right)=\frac{\lambda_{7}-\lambda_{8}}{-\lambda_{9}+\lambda_{7}}, \end{array} \end{array}$$

where $W=W_{1}\left(2\right)=W_{2}\left(1\right)$ and

$$\begin{array}{c} \lambda_{1}=\mathrm{erf}\left(\frac{a_{1}\;\left(S_{0}^{d}-1\right)}{\lambda_{0}}\right),\;\lambda_{2}=\mathrm{erf}\left(\frac{S_{0}^{d}\;a_{1}+\left(\rho -2\right)\;a_{1}-b_{1}\;\left(\rho -1\right)}{\lambda_{0}}\right),\;\lambda_{3}=\mathrm{erf}\left(\frac{S_{0}^{d}\;a_{1}-b_{1}}{\lambda_{0}}\right),\\ \lambda_{4}=\mathrm{erf}\left(\frac{\left(\left(b_{1}\;\varepsilon_{2}+\left(-b_{1}+a_{1}\right)\;\varepsilon_{1}-a_{1}\right)\;S_{0}^{d}-s_{1}\;\left(-1+\varepsilon_{2}\right)\right)\;\left(b_{1}-s_{1}\right)}{\lambda_{00}}\right),\\ \lambda_{5}=\mathrm{erf}\left(\frac{\left(b_{1}-s_{1}\right)\;\left(\left(\left(\varepsilon_{2}-\varepsilon_{1}\right)\;b_{1}+a_{1}\;\left(-1+\varepsilon_{1}\right)\right)\;S_{0}^{d}+\left(-1+\varepsilon_{2}\right)\;\left(\left(\rho -2\right)\;b_{1}-s_{1}\;\left(\rho -1\right)\right)\right)}{\lambda_{00}}\right),\\ \lambda_{6}=\mathrm{erf}(\frac{(\left(\left(\varepsilon_{2}-\varepsilon_{1}\right)\;b_{1}+a_{1}\;\left(-1+\varepsilon_{1}\right)\right)\;S_{0}^{d}-b_{1}\;\left(-1+\varepsilon_{2}\right)\;\left(b_{1}-s_{1}\right)}{\lambda_{00}}),\\ \lambda_{7}=\mathrm{erf}\left(\frac{\left(b_{1}\;\varepsilon_{2}+\left(-b_{1}+a_{1}\right)\varepsilon_{1}-a_{1}\right)\;S_{0}^{d}-b_{1}\;\left(-1+\varepsilon_{2}\right)}{2(-1+\varepsilon_{2})}\right),\\ \lambda_{8}=\mathrm{erf}\left(\frac{\left(b_{1}\;\varepsilon_{2}+\left(-b_{1}+a_{1}\right)\;\varepsilon_{1}-a_{1}\right)\;S_{0}^{d}+\left(-1+\varepsilon_{2}\right)\;\left(\left(\rho -2\right)\;b_{1}-s_{1}\;\left(\rho -1\right)\right)}{2(-1+\varepsilon_{2})}\right),\\ \lambda_{9}=\mathrm{erf}\left(\frac{\left(b_{1}\;\varepsilon_{2}+\left(-b_{1}+a_{1}\right)\;\varepsilon_{1}-a_{1}\right)\;S_{0}^{d}-s_{1}\;\left(-1+\varepsilon_{2}\right)}{2(-1+\varepsilon_{2})}\right),\\ \lambda_{0}=2\;\sqrt{\frac{1}{D_{W}}}\;D_{W},\;\lambda_{00}=2\;\sqrt{\frac{{\left(b_{1}-s_{1}\right)}^{2}}{D_{W}}}\;D_{W}\;\left(-1+\varepsilon_{2}\right). \end{array}$$

Equations (A17)–(A19) form a nonlinear system of algebraic equations for the unknowns $W,a_{1}$ and $b_{1}$ ,which can routinely be solved numerically. Equations (A20) and (A21) are used to formulate the initial conditions at Phase 1.1.

### Appendix B.2. Phase 2.1

With α=β and S−β≪1 for small time, we have from Equation (A2) $\partial_{\rho \;\rho }W_{2}∼0$ and $\partial_{\rho \;\rho }C_{2}∼0$ in the leading order. Furthermore, as $W_{2}\left(2,\tau \right)=1,$ we anticipate $\dot{\beta }=O\left(1\right)$ from the first in Equation (A10), and the second and third terms of Equation (A10) imply $\partial_{\rho }W_{2}\left(1,\tau \right)∼0$ and $\partial_{\rho }C_{2}\left(1,\tau \right)∼0$, hence $W_{2}∼1$ and $C_{2}∼1$ at leading order. We write

$$\begin{array}{cc} \beta \; & ∼\;S_{0}+b_{1}^{\left[1\right]}\tau +b_{2}^{\left[1\right]}\tau^{2},\\ S\; & ∼\;S_{0}+s_{1}^{\left[1\right]}\tau +s_{2}^{\left[1\right]}\tau^{2},\\ W_{2}\; & ∼\;1+W_{2_{1}}^{\left[1\right]}\left(\rho \right)\tau +W_{2_{2}}^{\left[1\right]}\left(\rho \right)\tau^{2},\\ C_{2}\; & ∼\;1+C_{2_{1}}^{\left[1\right]}\left(\rho \right)\tau +C_{2_{2}}^{\left[1\right]}\left(\rho \right)\tau^{2}. \end{array}$$

On substitution into Equations (A10) and (34), we obtain

$$\begin{array}{c} b_{1}^{\left[1\right]}=-\frac{\kappa }{\gamma_{0}},\;\;b_{2}^{\left[1\right]}=-\frac{\kappa }{\gamma_{0}}\;W_{2_{1}},\;\;s_{1}^{\left[1\right]}=\left(1-w_{\alpha }w_{\beta }\right)\;b_{1},\;\;s_{2}^{\left[1\right]}=\left(1-w_{\alpha }w_{\beta }\right)\;\left(\frac{b_{1}^{{\left[1\right]}^{2}}\;w_{\alpha }w_{\beta }\;d}{2}+b_{2}^{\left[1\right]}\right), \end{array}$$

where $W_{2}^{\left[1\right]}$ is defined below. For Equations (A2) and (A3), we have $\partial_{\rho \;\rho }W_{2_{1}}=0$ and $\partial_{\rho \;\rho }C_{2_{1}}=0$, and, using Equation (A10), we obtain $W_{2_{1}}^{\left[1\right]}$ and $C_{2_{1}}^{\left[1\right]}$ on integration yielding

(A22) $$\begin{array}{c} \begin{array}{c} W_{2}=1+\frac{\gamma_{1}w_{\alpha }b_{1}\left(s_{1}-b_{1}\right)\left(2-\rho \right)}{D_{W}\left(1-\varepsilon_{2}\right)}\;\tau ,\\ C_{2}=1+\frac{\gamma_{2}w_{\alpha }b_{1}\left(s_{1}-b_{1}\right)\left(2-\rho \right)}{\left(1-\varepsilon_{2}\right)}\;\tau , \end{array} \end{array}$$

as τ→0. From Equations (A2) and (A3), the next order equations are

$$\begin{array}{cc} \partial_{\rho \;\rho }W_{2_{2}} & =\frac{2\partial_{\rho \;\rho }W_{2_{1}}\left(s_{2}\;-\;b_{2}\right)}{\left(s_{1}-b_{1}\right)}\;+\;\frac{\left(s_{1}\;-\;b_{1}\right)}{D_{W}}\left[\left(b_{1}\;-\;s_{1}\right)\left(\rho \;-\;1\right)\;-\;b_{1}\;+\;\frac{\left(1\;-\;\varepsilon_{1}\right)a_{1}\;-\;\left(\varepsilon_{2}\;-\;\varepsilon_{1}\right)b_{1}}{\left(1-\varepsilon_{2}\right)}\;-\;\frac{dD_{W}}{S_{0}}\right]\partial_{\rho }W_{2_{1}},\\ \partial_{\rho \;\rho }C_{2_{2}} & =\frac{2\partial_{\rho \;\rho }C_{2_{1}}\left(s_{2}\;-\;b_{2}\right)}{\left(s_{1}-b_{1}\right)}\;+\;\left(s_{1}\;-\;b_{1}\right)\left[\left(b_{1}\;-\;s_{1}\right)\left(\rho \;-\;1\right)\;-\;b_{1}\;+\;\frac{\left(1\;-\;\varepsilon_{1}\right)a_{1}\;-\;\left(\varepsilon_{2}\;-\;\varepsilon_{1}\right)b_{1}}{\left(1\;-\;\varepsilon_{2}\right)}\;-\;\frac{d}{S_{0}}\right]\partial_{\rho }C_{2_{1}}, \end{array}$$

hence the advection terms come into play in the $\tau^{2}$ terms. We use the expansions for β,S and Equation (A22) for the initial conditions in Phase 2.1.

### Appendix B.3. Phase 2.2

This phase occurs after Phase 2.1 when α and β begin to separate at $\tau =T_{\alpha =\beta }$. The values of $\alpha =\beta ,S,W_{2}$ and $C_{2}$ at $\tau =T_{\alpha =\beta }$ are known from the numerical solution of Phase 2.1. For $0<\tau -T_{\alpha =\beta }\ll 1$ and 0<β−α≪1, we write

$$W_{1}∼W_{2}\left(1,\tau \right)+\left(\beta -\alpha \right)A\;\left(2-\rho \right)$$

and $\alpha =\alpha_{0}\left(\tau \right)+O\left(\tau -T_{\alpha =\beta }\right)$, then the first in Equation (A10) implies that

(A23) $$\begin{array}{c} \dot{\alpha_{0}}=-\frac{\kappa \;W_{2}^{2}\left(1,\tau \right)}{\gamma_{0}}, \end{array}$$

in leading order, implying from the first in Equation (A8) that

$$\begin{array}{c} -\left(1-\varepsilon_{1}\right)\;\frac{D_{W}}{\beta -\alpha }\;\partial_{\rho }W_{1}\left(1,\tau \right)∼2\;\kappa \;W_{2}^{2}\left(1,\tau \right), \end{array}$$

resulting with $A=2\kappa W_{2}^{2}\left(1,\tau \right)/\left(1-\varepsilon_{1}\right)$ and hence

(A24) $$\begin{array}{c} W_{1}∼W_{2}\left(1,\tau \right)+\left(\beta -\alpha \right)\frac{2\kappa W_{2}^{2}\left(1,\tau \right)}{\left(1-\varepsilon_{1}\right)}\left(2-\rho \right). \end{array}$$

For the initial part of Phase 2.2, Equations (A2) and (A3) are solved numerically until β−α reaches a given small tolerance, typically $\beta -\alpha =10^{-3}$, at $\tau =T_{\alpha =\beta }+\tau_{1}$, for example. Following this, the system for Phase 2.2 is solved in the usual fashion starting from time $T_{\alpha =\beta }+\tau_{1}$ using the updated solutions for $W_{2},C_{2},\alpha$ and β, and by using Equation (A24) for $W_{1}$.
